# Systematic Review and Dosage Analysis: Hyperbaric Oxygen Therapy Efficacy in Mild Traumatic Brain Injury Persistent Postconcussion Syndrome

**DOI:** 10.3389/fneur.2022.815056

**Published:** 2022-03-17

**Authors:** Paul G. Harch

**Affiliations:** Hyperbaric Medicine Unit, Section of Emergency Medicine, Department of Medicine, Louisiana State University Health Sciences Center, New Orleans, LA, United States

**Keywords:** traumatic, brain, injury, traumatic brain injury, hyperbaric oxygen, therapy, postconcussion, postconcussion syndrome

## Abstract

**Background:**

Mild traumatic brain injury results in over 15% of patients progressing to Persistent Postconcussion Syndrome, a condition with significant consequences and limited treatment options. Hyperbaric oxygen therapy has been applied to Persistent Postconcussion Syndrome with conflicting results based on its historical understanding/definition as a disease-specific therapy. This is a systematic review of the evidence for hyperbaric oxygen therapy (HBOT) in Persistent Postconcussion Syndrome using a dose-analysis that is based on the scientific definition of hyperbaric oxygen therapy as a dual-component drug composed of increased barometric pressure and hyperoxia.

**Methods:**

In this review, PubMed, CINAHL, and the Cochrane Systematic Review Database were searched from August 8–22, 2021 for all adult clinical studies published in English on hyperbaric oxygen therapy in mild traumatic brain injury Persistent Postconcussion Syndrome (symptoms present at least 3 months). Randomized trials and studies with symptomatic and/or cognitive outcomes were selected for final analysis. Randomized trials included those with no-treatment control groups or control groups defined by either the historical or scientific definition. Studies were analyzed according to the dose of oxygen and barometric pressure and classified as Levels 1–5 based on significant immediate post-treatment symptoms or cognitive outcomes compared to control groups. Levels of evidence classifications were made according to the Centre for Evidence-Based Medicine and a practice recommendation according to the American Society of Plastic Surgeons. Methodologic quality and bias were assessed according to the PEDro Scale.

**Results:**

Eleven studies were included: six randomized trials, one case-controlled study, one case series, and three case reports. Whether analyzed by oxygen, pressure, or composite oxygen and pressure dose of hyperbaric therapy statistically significant symptomatic and cognitive improvements or cognitive improvements alone were achieved for patients treated with 40 HBOTS at 1.5 atmospheres absolute (ATA) (four randomized trials). Symptoms were also improved with 30 treatments at 1.3 ATA air (one study), positive and negative results were obtained at 1.2 ATA air (one positive and one negative study), and negative results in one study at 2.4 ATA oxygen. All studies involved <75 subjects/study. Minimal bias was present in four randomized trials and greater bias in 2.

**Conclusion:**

In multiple randomized and randomized controlled studies HBOT at 1.5 ATA oxygen demonstrated statistically significant symptomatic and cognitive or cognitive improvements alone in patients with mild traumatic brain injury Persistent Postconcussion Syndrome. Positive and negative results occurred at lower and higher doses of oxygen and pressure. Increased pressure within a narrow range appears to be the more important effect than increased oxygen which is effective over a broad range. Improvements were greater when patients had comorbid Post Traumatic Stress Disorder. Despite small sample sizes, the 1.5 ATA HBOT studies meet the Centre for Evidence-Based Medicine Level 1 criteria and an American Society of Plastic Surgeons Class A Recommendation for HBOT treatment of mild traumatic brain injury persistent postconcussion syndrome.

## Introduction

This is a scientific review of the evidence for hyperbaric oxygen therapy treatment of non-penetrating mild traumatic brain injury (mTBI) (1) Persistent PostConcussion Syndrome (PPCS) (2). The conclusions and recommendations are based on the Centre for Evidence-Based Medicine (CEBM) Levels of Evidence (3) and the American Society of Plastic Surgeons Grade Practice Recommendation guidelines for clinical treatment (4).

TBI (5, 6) is a heterogeneous (7–27) diffuse physical injury to the brain that causes mechanical (7, 8, 28, 29) disruption of gray (22–24, 30, 31) and white (10, 15, 22, 23, 30, 32–35) matter, ischemia (36), hypoxia (29, 37, 38), edema (29, 39, 40), vasospasm (41, 42), release of neurochemicals (43, 44), and reperfusion injury (39, 45) and affects over 4.1 million people annually in the U.S. alone (46). These gray and white matter wounds in both blunt (10, 11) and blast TBI (22, 24) mature with time, resulting in the downstream synaptic loss (9, 23, 47), nerve cell loss (10, 11), and overall tissue loss (25–27, 48). This chronic tissue pathology is responsible for permanent postconcussive symptoms in over 15% of mTBI patients (49–51) and has been paradoxically designated a psychiatric condition, PPCS (2). Treatment has consisted of adaptive, stimulative, or accommodative approaches with limited evidence of efficacy (52–54). None of them address the biological repair of the underlying gray and white matter wounds.

Hyperbaric oxygen therapy (HBOT) has been historically defined as treatment with 100% oxygen at a minimum arbitrary pressure of 1.4 atmospheres absolute (ATA) (55, 56) for a narrow list of acute and chronic wound conditions (56). The U.S. Food and Drug Administration (FDA) has categorized HBOT as a prescription medical gas (oxygen) consisting of increased barometric pressure and hyperoxia (57). It has been scientifically defined as “a medical treatment that uses increased atmospheric pressure and increased oxygen as drugs by fully enclosing a person or animal in a pressure vessel and then adjusting the dose of the drugs to treat pathophysiologic processes of the diseases” (58). Based on this scientific definition HBOT can be appreciated as a treatment for common acute and chronic wound pathophysiology (56, 58–60) found in acute and chronic wound conditions (56, 58–62).

Hyperbaric oxygen therapy (HBOT) has been applied to chronic TBI wounds in animals and humans since 1989 (46, 63–98) with apparently conflicting results for mTBI (46, 66–89, 99–105). Various researchers have attributed the different results to mischaracterized sham groups/the effects of different doses of HBOT (46, 80, 99–105), design differences (106), (small sample size, dissimilar outcome measures/populations/sites/protocol adherence, non-equivalence of groups, selection bias) (73, 81), ritual experience (75), and placebo/Hawthorne effects (107). The conflict stems from control group selection based on the historical definition of HBOT (55) where the bioactivity of barometric pressure and <100% oxygen were dismissed. This systematic review of the science and literature on HBOT in mTBI PPCS analyzes the studies/data based on the scientific definition of HBOT (58) and the U.S. FDA's classification of HBOT as a dual-component drug (57). Recognizing that the HBOT studies consist of different doses of increased pressure and hyperoxia, the evidence is ranked according to the Evidence-Based Medicine hierarchy of evidence for treatment in clinical studies (3). The analysis demonstrates Level I evidence for the efficacy of 40 HBOTs at 1.5 ATA of oxygen (46, 75) in the treatment of blast or blunt mTBI/PPCS.

## Methods

This systematic review was reported in accordance with the Preferred Reporting Items for Systematic Reviews and Meta-Analyses (PRISMA) guidelines (108). The PRISMA checklist is attached as Supplementary Material.

### Search Method

In this review, PubMed, CINAHL, and the Cochrane Systematic Review Database were searched without filters or time limits on August 18, 8, and 8, 2021, respectively, for English language clinical articles using the terms hyperbaric oxygen, as well as traumatic brain injury, mild traumatic brain injury, postconcussion syndrome, or persistent postconcussion syndrome. Reference lists were reviewed for additional studies. Inclusion criteria consisted of studies with immediate post-treatment symptom or cognitive outcomes and: adult subjects 18–65 years old, persistent postconcussion syndrome (postconcussion symptoms lasting at least 3 months), one or more blast or blunt mTBIs, and treatment with HBOT. Symptomatic outcomes were those employing TBI symptom questionnaires; cognitive outcomes were computerized or manual cognitive tests. Affective measures, imaging, balance, eye-tracking, and sleep measures used as stand-alone outcomes were not included. The search process for inclusion and exclusion criteria was: first search (title screen), second search (abstract review of first search titles), third search (full-text articles of abstracts), fourth search (detailed review of full-text articles).

Manual data extraction was performed on the final selected articles from the abstracts, results, and conclusion sections of the articles and entered in Table 1. Numerical data included study publication year, design, number of subjects, gender, civilian or military status (active duty or veteran), education level, time from TBI to treatment, number of total lifetime TBIs, type of TBI (blast or blunt), percent of subjects with comorbid post-traumatic stress disorder (PTSD), traditional pressure/oxygen dose parameters, the total number of prescribed HBOTs in the protocol, and outcome instruments. Symptomatic and cognitive results of the studies were as stated in the abstracts and conclusions without numerical figures; their numerical data and calculated oxygen dose/study were entered in the text of the Section Results. Symptom outcomes for the final selected studies were averaged and analyzed by treatment pressure and total oxygen dose. All searches, screening, selection, data extraction, and analyses were performed by a single author. No automation tools were used for any part of this study. Missing data from some articles were obtained from companion articles of the same study on the same subjects. If not available from companion articles it was noted as “unidentified or unstated.” Studies were grouped by design for classification of evidence: randomized trials, cohort studies, case-controlled studies, case series, and case reports. Reviews and pooled data analyses were excluded.

**Table 1:** Retrieved analyzed studies and companion articles.

**References**	**Design**	**Number of subjects, Male (M), Female (F), Unidentified or Unstated (U)**	**Age (yrs.)**	**Status: Mi (Military), AD (Active Duty), V (Veteran), C (Civilian)**	**Educa-tion (yrs.)**	**Time from TBI to Rx (mos)**	**# TBIs**	**TBI: Blast (Bla)/ Blunt (Blu)**	**% PTSD**	**Pressure/Dose of HBOT**	**Number of HBOTs**	**Outcome instruments: Sx (symptoms), PEx (physical exam)**	**Results**
**Harch et al**. **(****66****)**	Case Report	1 M	25	Mi-V	15	36	7	Bla	100%	1.5 ATA O_2_/60 min. total dive time (TDT); monoplace chamber	39, twice/day (bid), 5d/week, 1 month	Sx, PEx, and SPECT pre and within one week post-HBOT	Improvement Sx, PEx, SPECT
**Wright et al**. **(****67****)**	Case Report	2 M	23 22	Mi-AD	U	8	1 1	Bla Bla	U	1.5 ATA O_2_/60 min. at depth; monoplace chamber	40, once/day (qd), 5d/week; 80 in two 40 treatment blocks separated by ~1 month break	Sx, ANAM, mood, and sleep pre and within 4–6 weeks post HBOT (time deduced from testing dates).	Improvement Sx, ANAM, mood, sleep
**Harch et al**. **(****68****)**	Prospective Case Series [included in Harch et al. (80)]	16 M	30	Mi-8 AD, 8 V	12.9	33.6	2.7	Bla	100% (Avg. PCL= 67)	1.5 ATA O_2_/60 min TDT; monoplace chamber	40, bid, 5d/week/1 month	21 neuropsychological, affective, Quality of Life, self-assess tests, Sx, PEx, SPECT pre and within one week after HBOT with phone questionnaire followup at 6 months post HBOT	Significant improvement Sx, PEx, 15/21 outcome instruments, and SPECT. Significant worsening 1/21 instruments
**Wolf et al**. **(****69****)-Air Force funded**	RCT, double-blind	50 (48 M, 2 F)	28.3	Mi-AD	Slightly > 12	3-71	3.4	Bla (33), Blu (8), both (9)	U	1.3 ATA air vs. 2.4 ATA O_2._ both for 90 min at depth with two 10 min air breaks for the O_2_ group. The 1.3 Air group slowly drifted to 1.2 ATA over the course of the treatment; multiplace chamber	30, qd, 5d/week for 6 weeks	PCL-M and ImPACT Sx questionnaires pre, weekly, and 6 weeks after intervention.	Significant within group improvement on PCL-M and ImPACT, but no significant differences between groups.
**Boussi-Gross et al**. **(****70****)**	RCT, cross-over, single-blind	56 (24 M, 32 F)	44	C	15.3	33	1	Blu	U	1.5 ATA O_2_ for 60 min at depth; multiplace chamber.	40, qd, 5d/week for 8 weeks	Mindstreams computerized cognitive test battery, Quality of Life questionnaire, SPECT pre and 1–3 weeks “after the HBOT protocol.”	Significant improvement in cognitive function and Quality of Life in both groups post HBOT, none during control. Increased brain activity on SPECT after HBOT compared to controls, in agreement with cognitive improve.
**Cifu et al**. **(****71****)-Defense Advanced Research Projects Agency (DARPA) funded**	RCT, double-blind	61 M	23.2	Mi-AD	U	8.5	1 (75%), >1 (25%)	Bla	U	0.21, 1.5, and 2.0 ATA O_2_ at 2 ATA pressure/60 min at depth; multiplace chamber	40, qd, 5d/week, within 10 weeks	PCS sand PTSD symptom questionnaires (Rivermead and PCL-M) pre and immediately post intervention.	No between group differences Rivermead and PCL. Significant improvement PCL in 2.0 ATA O_2_ group.
Walker et al. (72)-DARPA funded	RCT [part of Cifu et al. (71)]	61 M	23.2	Mi-AD	U	8.5	1 (75%), >1 (25%)	Bla	U	0.21, 1.5, and 2.0 ATA O_2_ at 2 ATA pressure/60 min at depth; multiplace chamber	40, qd, 5d/week, within 10 weeks	Battery of 55 cognitive, psycho-motor, and balance tests pre and within 1 week post intervention.	“No beneficial effect 1.5 or 2.0 ATA O_2_ compared to sham.”
Cifu et al. (73)-DARPA funded	RCT [part of Cifu et al. (71)]	61 M	23.3	Mi-AD	U	8.5	1 (75%), >1 (25%)	Bla	U	0.21, 1.5, and 2.0 ATA O_2_ at 2 ATA pressure/60 min at depth; multiplace chamber	40, qd, 5d/week, within 10 weeks	17 cognitive, psychomotor, functional, Quality of Life tests pre, within 1 week, and 3 months post last HBOT.	“No significant time by intervention interaction for any outcome measure.”
Cifu et al. (74)-DARPA funded	RCT [part of Cifu et al. (71)]	61 M	23.3	Mi-AD	U	8.5	1 (75%), >1 (25%)	Bla	U	0.21, 1.5, and 2.0 ATA O_2_ at 2 ATA pressure/60 min at depth; multiplace chamber	40, qd, 5d/week, within 10 weeks	Computerized eye tracking measurement of fixation, saccades, and smooth pursuit pre, immediately, and 3 months post intervention.	“No significant between group effects or 1.5 or 2.0 ATA O_2_ effects vs. sham.”
**Miller et al**. **(****75****)-HOPPS (Hyperbaric Oxygen Therapy for Persistent Post-concussive Symptoms)-Army funded**	RCT, double-blind	72 (69 M, 3 F)	31.4	Mi-AD	2/3rds with > 12 years	22.9	3.1	U	65%, (47/72) Avg PCL-C = 51.3	1.2 ATA air or 1.5 ATA O_2_/50 min at depth vs. No chamber treatment;multiplace chamber	40, qd, within 10 weeks	RPQ-3, 13, and 16, NSI, ANAM pre, after 20 and 40 treatments or 10 weeks; PCL-C, depression, anxiety, pain, sleep, Quality of Life pre and after 40 treatments or 10 weeks.	No difference between air and O_2_ treatments; both groups Significantly improved compared to no hyperbaric treatment
Wolf et al. (76)-Air Force funded	RCT [part of Wolf et al. (69)-Air Force]	50 (48 M, 2 F)	28.3	Mi-AD	Slightly > 12	3–71	3.4	Bla (33), Blu (8), both (9)	U	1.3 ATA air vs. 2.4 ATA O_2._ both for 90 min at depth with two 10 min air breaks. The 1.3 Air group drifted to 1.2 ATA slowly over the course of the treatment; multiplace chamber	30, qd, 5d/week for 6 weeks	ImPACT, ANAM, ToVA, PCL-M, pre, weekly, and 6 weeks after intervention.	Significant improve. ImPACT visual memory and time processing, ANAM code substitution recall, match to sample, and simple reaction, PCL-M within both groups, no significant between group findings. Sub-groups identified.
Churchill et al. (77)-HOPPS	RCT [Part of Miller et al. (75)-HOPPS]	64 of 72 (61 M, 3 F)	33	Mi-AD	Not stated for the 64; 2/3rds with > 12 years for the 72 subjects	24	4	U	U for the 64, but 65% (47/72). Avg PCL-C = 51.3	1.2 ATA air or 1.5 ATA O_2_/60 min at depth vs. No chamber treatment; multiplace chamber	40, qd, within 10 weeks	Simple Reaction Time (SRT) and Procedural Reaction Time (PRT) of ANAM pre, at midpoint (6 weeks), and within 1 month post intervention (13 weeks).	SRT worsened for local care group. No significant differences between 3 groups over time
Skipper et al. (78)-DARPA and HOPPS	RCT-2 combined studies [Cifu et al. (71)-DARPA and Miller et al. (75)-HOPPS]	40 (All M)	28.1	Mi-AD	57.5% with “some college or more”	U	87.5% with multiple	U	U for the 40 as a group	0.21, 1.5, and 2.0 ATA O_2_ at 2 ATA pressure/60 min at depth, and 1.2 ATA air or 1.5 ATA O_2_/60 min at depth vs. No chamber treatment; multiplace chambers.	40, qd within 10 weeks, both studies	PCS, PTSD, anxiety, depression, and Quality of Life average 39 months post treatment	DARPA: PCS scores worse 1.5 ATA O_2_, improved 0.21 and 2.0 ATA O_2_ groups. HOPPS: PCS scores worsened in all groups.
Shandley et al. (79)-Air Force funded	RCT [part of Wolf et al. (69)-Air Force]	28 (U M/F)	U	Mi-AD	U	U	U	U	U	1.3 ATA air vs. 2.4 ATA O_2._ both for 90 min at depth with two 10 min air breaks. The 1.3 Air group drifted to 1.2 ATA slowly over the course of the treatment; multiplace chamber	30, qd, 5d/week for 6 weeks	ImPACT, BrainCheckers, PCL-M pre and post 5, 10, 15, 20, 25, 30 HBOTs and at 6 week followup. Stem cells from peripheral blood (flow cytometry) pre, post 15 and 30 HBOTs and at 6 week followup.	HBOT at 2.4 ATA correlated with stem cell mobilization and increased cognitive performance
**Harch et al**. **(****80****)**	Case-Controlled (Partial; imaging control group of population normals)	30 (28 M, 2 F)	30.3	Mi-(11 AD, 19 V)	13.1	40.2	3.5	Bla	77% (23/30); Avg. PCL= 63.4	1.5 ATA O_2_/60 min TDT; monoplace chamber	40, bid, 5d/week/1 month	21 neuropsych, affective, Quality of Life, self-assessment tests, Sx, PEx, pre and within 1 week after HBOT with phone questionnaire followup at 6 months post HBOT. SPECT pre, after 1^st^ and 40th HBOTs.	Significant improvement Sx, neurol PEx, IQ, memoy, attention, dominant hand motor speed and dexterity, Quality of Life, anxiety, PTSD, depression, suicidal ideation, psychoactive medications. Further Sx improve at 6 months. Significant SPECT improvement after 1 and 40 HBOTs.
**Weaver et al**. **(****81****)-BIMA (Brain Injury Mechanisms of Action) DoD funded**	RCT, double-blind	71 (70 M, 1 F)	32.8	Mi-(68 AD, 3V)	82% “some college or more”	25.6	3.6	Bla (32%), Blu (20%), Com-bination of Bla and Blu (48%)	49% (avg. = 44.9)	1.2 ATA air or 1.5 ATA O_2_/60 min at depth; multiplace chamber	40, qd, Monday to Friday over 12 weeks	Sx, Quality of Life, neuropsychological, neurological, EEG, sleep, auditory, vestibular, autonomnic, visual, neuroimaging, and laboratory testing pre, at 13 weeks, and 6 months post randomization; 12 month post randomization online/phone questionnaires.	Significant improvement NSI and PTSD Sx in HBOT group, more pronounced for those with PTSD with regression at 3 and 9 months post treatment. HBOT improved some cog processing speed and sleep measures. Pts. With PTSD and HBOT improved functional balance and reduced vestibular complaints at 13 weeks.
Walker et al. (82) BIMA-DoD funded	RCT [Part of Weaver et al. (81)-BIMA]	71 (70 M, 1 F); 75 healthy volunteer (58 M, 17 F)	32.8; 39.3-Volun-teers	Mi-(68 AD, 3V); Mi-(1-AD, 21-V), 53-C	82%; 92% “some college or more”	25.6	3.6	Bla (32%), Blu (20%), Combina-tion of Bla and Blu (48%)	49% (avg. = 44.9)	1.2 ATA air or 1.5 ATA O_2_/60 min at depth; multiplace chamber	40, qd, Monday to Friday over 12 weeks	Pittsburgh Sleep Quality Index (PSQI), sleep diary, screen for obstructive sleep apnea, restless leg syndrome, and cataplexy; objective actigraphic measures of sleep-wake. All pre, 13 weeks, and 6 months post randomization.	Sleep quality by self-reports signifcantly abnormal compared to normals (70% obstructive sleep apnea risk). Significant improvement PSQI (5/8 measures at 13 weeks and 2/8 at 6 months) in HBO group compared to air group. No changes in other measures.
Meehan et al. (83)-BIMA-DoD funded	RCT [Part of Weaver et al. (81)- BIMA]	71 (70 M, 1 F); 75 healthy volunteer (58 M, 17 F)	32.8; 39.3-Volun-teers	Mi-(68 AD, 3V); Mi-(1-AD, 21-V), 53-C	82%; 92% “some college or more”	25.6	3.6	Bla (32%), Blu (20%), Combina-tion of Bla, and Blu (48%)	49% (avg. = 44.9)	1.2 ATA air or 1.5 ATA O_2_/60 min at depth; multiplace chamber	40, qd, Monday to Friday over 12 weeks	Dynamic posturography, vestibular evoked myo-genic potentials, tandem gait, Romberg, Sharpened Romberg, Berg Balance Scale, Beck Anxiety Inventory, PTSD Checklist-Civilian, DSM-IV PTSD Module, Center for Epidemiologic Study Depression Scale: all at baseline, 13 weeks, and 6 months post randomization.	mTBI cohort worse than healthy volunteers on balance and gait measures and affective symptoms. Significant improvement postural control favored HBOT, but were “minimal.” Those with affective Sx, especially PTSD, had the most improvement in postural control and otolith function after HBOT.
**Mozayeni et al**. **(****84****)**	Prospective Case Series	32 (29 M, 3 F)	30.5	Mi-(7 AD,12 V), C-13	U	114	U	Bla (47%), Blu (53%)	22% (7/32)	1.5 ATA O_2_ for 45 min (monoplace) or 50 min (multiplace) at depth.	40, qd, 5d/wk, with option for 20 or 40 additional HBOTs for residual symptoms	ANAM and CNS Vital Signs computerized cognitive test batteries pre, post 40, 60, and 80 treatments.	Improvement in 13/17 neurocognitive and 8/8 mood measures. More than 40 HBOTs, blast TBI, less delay to treatment, and patients with PTSD showed greater improvement
Hart et al. (86) BIMA	RCT [Part of Weaver et al. (81)-BIMA]	42 (41 M, 1 F)	33.8	Mi-(40 AD, 2 V)	U	26.1	3.7	Bla (31%), Blu (21%), comina-tion (48%)	52%	1.2 ATA air or 1.5 ATA O_2_/60 min at depth; multiplace chamber	40, qd, Monday to Friday over 12 weeks	PPCS, PTSD, Quality of Life, pain, depression, anxiety, alcohol use at 24 months (40 subjects), 36 months (14 subjects) post study	No significant differences between groups at 24 and 36 months and mean scores near pre-intervention values.
Hart et al. (87) Air Force, DARPA, HOPPS, BIMA	RCT-4 pooled DoD studies: Wolf et al. (69)-Air Force, Cifu et al. (71)-DARPA, Miller et al. (75)-HOPPS, and Weaver et al. (81)-BIMA	254-DoD (248 M,6 F); 135-3 other studies	29.3	Mi-AD	“Some college or more” = 58.8%; high school grad. = 41.2%	21.7	Avg. for 3 studies = 3.4, 4th study (Cifu) 75% with >1 TBI.	For 3 studies: 116 blast, 22 blunt, 43 both; not reporter for Miller et al. (75)	43% by PCL, 58% by Clin. Inter-view	Above: Wolf (69) Cifu et al. (71) Miller et al. (75) Weaver et al. (81). 125 HBOT, 106 “sham,” 23 local care	DoD: 40 [three studies (71, 75, 81)] and 30 (Air Force study) (69)	PCS, PTSD, and neuropsychological measures for all studies	Trend of improvement HBOT for PCS and PTSD Sx, verbal memory. Dose-response to increasing O_2_ pressure with greater effect with PTSD. Direction of results consistent with other studies.
Wetzel et al. (88) BIMA	RCT [Part of Weaver et al. (81)-BIMA]	71 (70 M, 1 F); 75 healthy volunteer (58 M, 17 F)	32.8; 39	Mi-(68 AD, 3V); Mi-(1-AD, 21-V, 53-C)	82%; 92% “some college or more”	25.6	3.6	Bla (32%), Blu (20%), Combina-tion of Bla and Blu (48%)	49% (avg. = 44.9)	1.2 ATA air or 1.5 ATA O_2_/60 min at depth; multiplace chamber	40, qd, Monday to Friday over 12 weeks	Eye movement tracking for saccadic and smooth pursuit pre, at 13 weeks and 6 months post randomization.	No between group, but within group improvement at all time points. Normals and all BIMA subjects no longer significantly different at 13 weeks and 6 months.
**Shytle et al**. **(****89****)**	Case Report	3 (2 M, 1 F)	27.3	Mi-2 V, 1-U	1 college graduate, 2-U	48- (1), U-(2)	1: “daily IEDs + burn pit;” 1-“numerous IEDs;” 1-Blu + at least 3-4 Bla	Bla-2, Blu + Bla-1	100%	1.75 ATA O_2_; 1.5 ATA O_2_; 1.5 ATA O_2_, All at 60 min TDT; monoplace chamber.	20: qd, 5d/week; 30: bid, 5d/week; 35: bid, 5d/week x 25 + qd, 5d/week x 10	“Computer-assisted assessments”-(type not specified)-pre/post for all 3. 1 patient also had pre/post CNS Vital Signs, Inc. and 1 had pre/post NeuroPsych^TM^	“Improvements on parameters within neuro-cognitive domains,” symptoms, reduction in suicidal-related symptoms.
**Harch et al**. **(****46****)**	RCT, cross-over, single blind	50 (21 M, 29 F)	42.5	C-41, Mi-9 (1-AD, 8-V)	14.0 (HBOT); 15.6 (Control)	55.2	3.9	Blu-45, Bla-5	0%	Control (no Rx) vs. 1.5 ATA O_2_/60 min TDT; monoplace chamber	40, qd, 5d/week	Symptom, neuro-psychological, and psychological testing pre/post and 2-month after last HBOT. Weekly NSI during treatment for both groups.	Significant improvement PPCS and PTSD symptoms, ANAM, memory, depression, anxiety, sleep, quality of life vs. control. Crossover experienced same improvement after HBOT

*Final analysis studies in bold*.

### Classification of Evidence/Analysis of Studies

Studies were classified as Level 1–5 according to CEBM guidelines (3). Classifications were based on significant immediate post-treatment symptom and/or cognitive outcomes in treatment groups vs. no-treatment control groups or lower doses of HBOT and compared according to the individual pressure, total oxygen, combined hyperbaric pressure and total oxygen doses of HBOT. The pressure was measured in atmospheres absolute (ATA) and oxygen in atmosphere-minutes (AMs). AM oxygen dose was the total cumulative oxygen dose over the entire treatment course per group for oxygen in excess of room air. It was the sum of the product of the atmospheric pressure times the FiO_2_ times the number of minutes for each phase of every hyperbaric treatment (compression, at depth, and decompression phase), multiplied times the total number of treatments. Constant compression and decompression rates were assumed. The average pressure from the surface to depth (compression) and depth to the surface (decompression) was multiplied by the FiO_2_ of the breathing gas for these phases. A linear increase in FiO_2_ and ~90% FiO_2_ was assumed by 8 min of compression (98) for protocols in monoplace (single-person) chambers that compressed with 100% oxygen. Practice Grade Recommendation was determined from the levels of evidence, according to the American Society of Plastic Surgeons grading system in Burns et al. (4).

### Methodologic Quality and Risk of Bias Assessment

The physiotherapy evidence database (PEDro) scale (109) was used to assess the methodologic quality/risk of bias of the randomized trials included in the final analysis. Randomized trials are given a cumulative score of 0–10 based on individual scoring of 10/11 items (the first, eligibility criteria, is omitted from the score because it is an external validity item), using 1 point for “present,” and 0 points for “absent.” The 10 items beginning with #2 are: (2) random allocation, (3) concealed allocation, (4) groups similar at baseline, (5) subject blinding, (6) therapist blinding, (7) assessor blinding, (8) 1 key outcome for > 85% of subjects, (9) 1 key outcome: intention-to-treat analysis, (10) 1 key outcome between-group statistical comparison, and (11) 1 key outcome point measurements and variability. Scoring was performed by the single author based on stated scoring items in the text of each article. Additional reporting bias and conflict of interest were noted for investigators who were employees of the funding source. All items were scored as 0 if they were not mentioned. Attempts were made to contact the authors of the studies to resolve the scoring on allocation concealment. Quality of studies (“poor, fair, good, excellent”) was judged according to the scoring legend of Cashin et al. (110).

## Results

**Figure 1:**
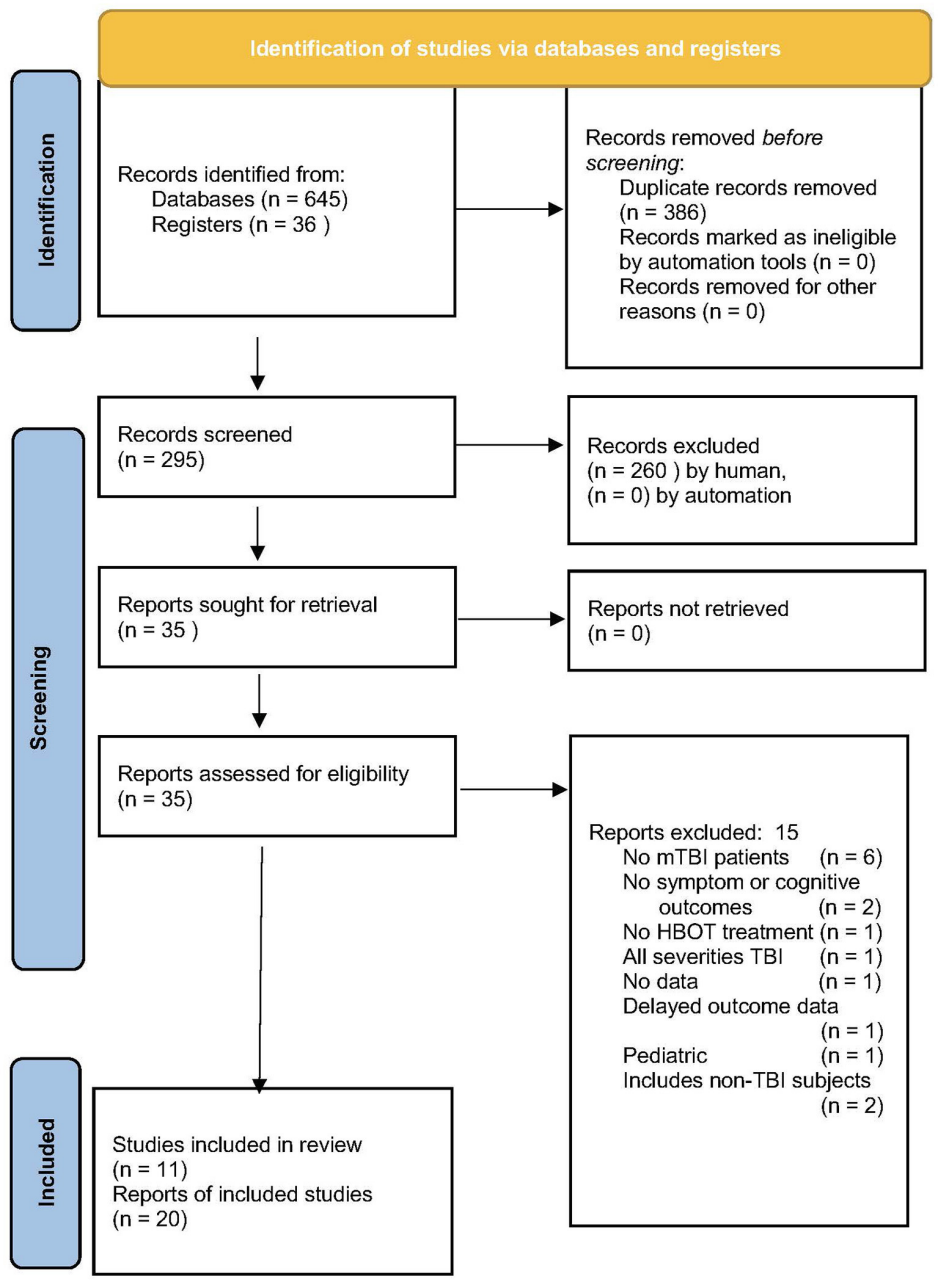
PRISMA flow chart of article search and retrieval (108).

In total, 681 articles were searched, 11 studies were included in the final analysis (Figure 1). The largest exclusion (57%) was for duplicate articles and the second-largest exclusion was by title (38%). An example of the third search was the Ma et al. study (111) on HBOT for firefighters with lifetime TBI and chronic emotional distress. The study was excluded due to the primary outcome of regional cerebral blood flow and the absence of symptomatic or cognitive outcomes.

The 11 studies that met the criteria for inclusion are listed in Table 1 (along with companion articles on non-primary outcomes). The 11 studies are: three case reports (66, 67, 89), a prospective case-controlled series reported in two publications (68, 80), a prospective case series (84), and six randomized trials (46, 69–71, 75, 81) reported in 19 publications (46, 69–79, 81–83, 85–88). Three of the randomized trials contained control groups (46, 70, 75), two with a crossover design (46, 70), and the other three were comparative dosing studies (69, 71, 81). Four of the randomized trials were performed by the U.S. Department of Defense (DoD) (69, 71, 75, 81) and were reported in multiple publications featuring different outcome instruments or follow-up periods [Wolf et al. (69) in Wolf et al. (76) and Shandley et al. (79); Cifu et al. (71) in Walker et al. (72) and Cifu et al. (73, 74); Miller et al. (75) in Churchill et al. (77); Weaver et al. (81) in Walker et al. (82), Meehan et al. (83), Hart et al. (86), and Wetzel et al. (88), pooled data analyses [Skipper et al. (78), Hart et al. (87)], and adverse events and blinding in Churchill et al. (85). Analysis was conducted on the three case reports (66, 67, 89), the case-controlled series (80), the case series (84), and six randomized trials (46, 69–71, 75, 81).

The case reports consist of Harch et al. (66), Wright et al. (67), and Shytle et al. (89). Harch et al. (66) reported symptomatic, neurological physical exam, and brain blood flow imaging improvements in an mTBI/PPCS veteran 3 years after the last of seven blast-induced combat mTBIs using forty 1.5 ATA oxygen HBOTs in 1 month (3,420 AMs oxygen). Wright et al. (67) demonstrated improvement of post-injury ANAM deficits, symptoms, mood, and sleep complaints in two military members with 40 and 80 1.5 ATA oxygen HBOTs (3,990 and 7,980 AMs oxygen) delivered 8 months after single blast-induced mTBIs. Shytle et al. (89) reported three cases with limited documentation of PPCS. In the first case mTBI/PPCS PTSD symptoms and computerized cognitive and mood test deficits/abnormalities improved with 20 HBOTs at 1.75 ATA oxygen (1,992 AMs oxygen). The second case experienced an improvement in symptoms and computerized affective and cognitive measures with 30 HBOTs at 1.5 ATA oxygen (2,544 AMs oxygen), and the third patient improved symptoms and computerized cognitive assessments with 35 HBOTs at 1.5 ATA oxygen (2,964 AMs oxygen). In summary, six out of six cases demonstrated symptomatic improvement, and five out of five demonstrated computerized cognitive testing improvement with HBOT treatment at 1.5 ATA or 1.75 ATA oxygen (one patient). Statistics were not reported for any patients in any of the case reports.

In the prospective case-controlled series, Harch et al. (68, 80) reported the first 16 (68) and then all 30 (80) military subjects with mTBI/PPCS and PTSD. The first 16 subjects (68) which were not case-controlled experienced significant improvement in self-reported symptoms, PPCS and PTSD symptom questionnaires (RPQ total, post-treatment minus pre-treatment: −15.6 ± 12.8, CI: −22.7 to −8.5, *p* = 0.0002), abnormal neurological exam, 7/13 neuropsychologist-administered cognitive tests, two neuropsychologist-administered affective instruments, and two quality of life measures after 40 HBOTs, and quantitative texture and statistical parametric mapping analysis of SPECT brain blood flow imaging after the first and 40th HBOT at 1.5 ATA oxygen (3,335 AMs oxygen). Statistical parametric mapping analysis revealed significant increases in brain blood flow in 85 regions of the brain, almost exclusively white matter, and in the hippocampi, that were consistent with the statistically and clinically significant increases in neuropsychologically measured working and delayed memory. The SPECT findings were different from and inconsistent with placebo SPECT brain blood flow studies. This non-controlled portion of the case-controlled study concluded that the significant symptomatic, cognitive, and SPECT improvements could not be explained by placebo effects.

The study design was changed at the midpoint of Harch et al. (68) to include a matched group of imaging controls for all 30 subjects and the full cohort of 30 subjects, 27 with comorbid PTSD, was reported as a case-controlled study in Harch et al. (80). Subjects had sustained 3.5 previous TBIs and were treated with 40 HBOTs at 1.5 ATA oxygen (3,420 AMs oxygen) 3.35 years post index TBI. Their PTSD symptom scores were 32% higher (63.4) than subjects in the four DoD studies [49.5 (69), 46.5 (71), 51.3 (75), 44.9 (81); average 48.1]. The 30 subjects experienced near-identical improvements to the first 16 subjects with statistically stronger *p*-values [RPQ total, post-treatment minus pre-treatment: −13.5 ± 10.4, CI: −17.4 to −9.6, *p* < 0.001 (actual *p* value was less, but the cutoff of 0.001 was used for this publication)], a significant reduction in suicidal ideation, and reduction in psychoactive medication use. SPECT brain imaging was abnormal in subjects compared to a matched cohort of population normals and normalized after HBOT in 75% of abnormal regions.

The prospective case series, Mozayeni et al. (84), reported outcomes on 32 military and civilian subjects treated 9.5 years after mTBI with 40–82 treatments (average 55.8) at 1.5 ATA oxygen (4,326 AMs oxygen). Mood changes and two computerized neurocognitive test batteries (ANAM4 and CNS Vital Signs) were administered; TBI symptom questionnaires were not used. Significant improvement was demonstrated on 8/8 Automated Neuropsychological Assessment Metrics (ANAM) mood, 6/7 ANAM neurocognitive, and 7/10 CNS Vital Signs neurocognitive measures. Improved outcomes were associated with shorter delays to treatment, younger age, military status, and increased numbers of HBOTs. In summary, both the prospective case-controlled and case series demonstrated multi-domain statistically significant outcomes/benefits of HBOT at a dose of 1.5 ATA oxygen with one study (80) showing simultaneous and correlative improvements in brain blood flow deficits compared to a group of untreated population normals.

The six randomized trials (46, 69–71, 75, 81) are separated into three comparative dosing studies (69, 71, 81) and three randomized controlled trials (46, 70, 75) based on the understanding of HBOT as a dual-component drug therapy consisting of increased barometric pressure and hyperoxia (46, 57, 58, 80, 99–101, 112). In each of the comparative dosing studies (69, 71, 81) and one of the controlled studies (75), the lower pressure compressed air groups (69, 75, 81) or normoxic oxygen group (71) were erroneously characterized as sham controls because the pressure/dose and FiO_2_ of HBOT were less than the historically defined arbitrary limit of 1.4 ATA 100% oxygen (55, 56). The inclusion of increased pressure and hyperoxia in these sham controls, the two bioactive components of hyperbaric oxygen treatment, precludes their characterization as shams; they are additional doses of hyperbaric therapy and hence classified as comparative dosing studies (46, 80, 99–101, 112).

**Figure 2:**
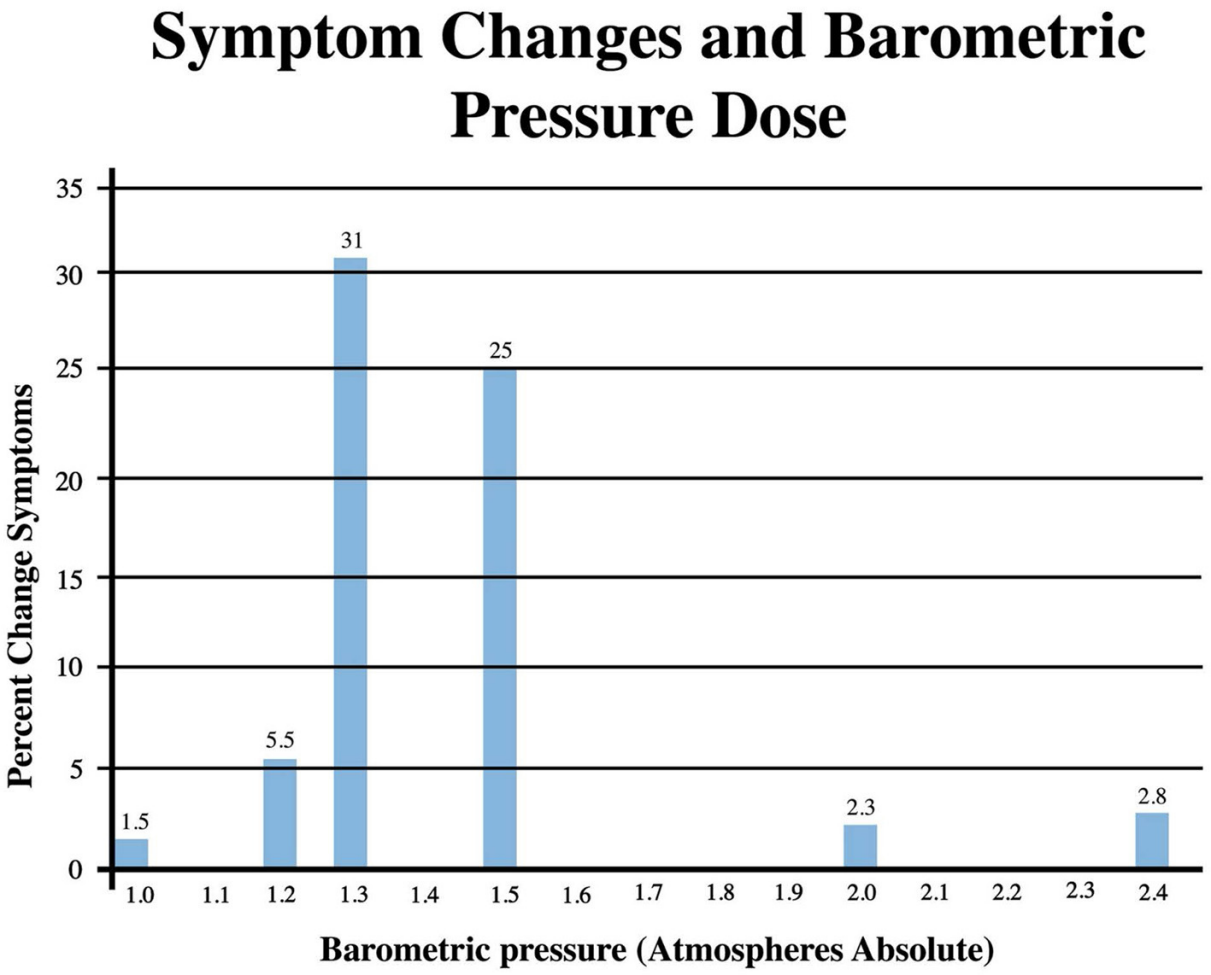
Symptom Improvements from Table 2 vs. pressure dose for HBOT mTBI PPCS studies. Symptom percent values represent average percent improvement in symptoms at a given pressure in Table 2, averaging the three different instrument (ImPACT, NSI, and RPQ) symptom outcomes.

The first of the comparative dosing studies was conducted by Wolf et al. (69) and randomized 50 military subjects 3–71 months after an average of 3.4 mTBIs to 30 1.3 ATA air or 2.4 ATA oxygen HBOTs (1,002 and 6,900 AMs oxygen, respectively). The authors found 6-week post-treatment symptom reductions (improvements) of 13% in the HBOT group and 41% in the 1.3 ATA air group with 10 subtests improved on the Immediate Post-Concussion Assessment and Cognitive Testing (ImPACT) symptom testing for the 1.3 ATA air group vs. two subsets in the 2.4 ATA oxygen group, but no within or between-group change score statistics were performed on this data. For the combined ImPACT symptom and cognitive total score 6-weeks post-treatment there were statistically significant reductions (improvements) in both groups [12% in the HBO_2_ group and 31% in the air group: control group (*t* = 3.76, *p* = 0.001) and the HBO_2_ group (*t* = 3.9, *p* = 0.001), but no statistical analysis was performed on the between-group improvements. Similarly, there were statistically significant within-group improvements in 6-weeks post-treatment PTSD symptoms, but no change score comparison between the two groups. Rather, it was stated that “Difference testing between the sham-control and HBO_2_ groups did not reveal any significant differences on the PCL-M composite mean score (*t* = −0.205, *p* = 0.84) or on the ImPACT total mean score (*t* = −0.943, *p* = 0.35) at any time (Figures 1, 2), including at 6 weeks post-exposure (Table 1).” In a subset analysis of this study Scorza et al. (113) reported that the mTBI/PPCS subjects without PTSD demonstrated a “trend toward harm” (worsening of scores) on the ImPACT symptom score in the 2.4 ATA oxygen group. This indicates a larger positive effect on 2.4 ATA subjects with comorbid PTSD and a negative effect on PPCS (the target of the study) without PTSD. Amplification of HBOT-induced PPCS benefit by comorbid PTSD was found in multiple studies (68, 80, 81, 87). The negative finding of Scorza (113) and corresponding 2.4 ATA oxygen group composite (symptoms and cognition) ImPACT score trajectory [Figure 2 in Wolf et al. (69) and Figure 4 in Harch et al. (46)] have been argued as a toxic/overdose effect of oxygen in the 2.4 ATA oxygen group (46, 80, 99).

The resulting cognitive outcomes in the study of Wolf et al. (69) were published in Wolf et al. (76) and showed significant within-group improvement for both groups, but no statistically significant between-group improvements for cognitive measures as well as PTSD symptoms. Curiously, there are no data presented in the paper. Rather, a derived metric, the “relative risk of improvement” (RROI) on subset historical features (e.g., 3 concussions, single event <2 years old, <1-year post-injury, blast injury, etc.) was reported. The article concluded that “Subgroup analysis of cognitive changes and Post-Traumatic CheckList Military results regarding PTSD demonstrated a relative risk of improvement using 2.4 atm abs hyperbaric oxygen.” While true for highly specific subsets of the 2.4 ATA oxygen group this conclusion is confusing and misleading, implying that HBOT at 2.4 is efficacious for PPCS. The “data” tables actually show that 60% of the RROI entries favored the 1.3 ATA air group and only 40% favored the 2.4 ATA oxygen group, consistent with the symptom data. The companion article (79), reporting cognitive improvements and stem cell mobilization, also used derived correlational data and is similarly misleading. A positive correlation was claimed between stem cell mobilization and cognitive improvements in the 2.4 ATA oxygen group, implying that stem cell mobilization was responsible for cognitive improvements in subsets of this group. This claim and implication are inconsistent with the symptom data/analysis of Scorza et al. (113) and the 60/40% air group subset cognitive dominance, and lack evidence of stem cell migration/implantation in the brain. Wolf et al. (76) acknowledge the finding of Scorza et al. (113) of significant improvement of subjects with co-morbid PTSD but omit the major finding that the 2.4 ATA oxygen dose demonstrated a “trend toward harm” in PPCS without PTSD (113). At the same time, it is known that stem cells are mobilized into the circulation of humans at 2.0 and 2.5 ATA oxygen (114) in the absence of PPCS and PTSD and that stem cells are stimulated to proliferate and differentiate at sites of injury in the brain due to HBOT (115–117), but there is no evidence of deposition of circulating stem cells in brain tissue in the Wolf et al. study (69, 76, 79). More likely, the circulating stem cells had nothing to do with the improvement in cognition in the PPCS PTSD group since cognitive and symptom improvements were favored in the air pressurization group that had no evidence of stem cell mobilization. In summary, these cognitive outcome reports (76, 79) do not report actual data, appear to be misleading, and the conclusions of the study, cognitive improvements in the 2.4 ATA oxygen group vs. the air group, are internally inconsistent and inconsistent with their own analysis (113) and the PPCS symptom data in Wolf et al. (69).

The second comparative dosing study (71) was reported in four publications (71–74) that randomized 61 military subjects 8.5 months post mTBI to one of three 40 treatment 2.0 ATA pressure groups with either 0.21, 1.5, or 2.0 ATA oxygen (76, 3,720, and 4,860 AMs oxygen). In the first publication (71) there were no significant within-group improvements (post minus pre mean change scores: “sham,” 0.05, *p* = 0.98; 1.5 ATA oxygen, 1.24, *p* = 0.61; 2 ATA oxygen, −3.77, *p* = 0.19) on the TBI Rivermead Postconcussion Questionnaire (RPQ) at 10 weeks post first HBOT (immediate/one-week post-treatment). Similar to Wolf et al. (69), no between-group change scores were statistically analyzed. Rather, one-way ANOVAs were performed on the pre-treatment mean RPQs for the three groups and then on the post-treatment mean RPQs. Identical analyses were performed on the PCL-M for PTSD symptoms; the 2 ATA oxygen group experienced the only significant finding in the study, a significant decrease in PTSD symptoms. The second publication (72) reported cognitive and psychomotor outcomes 1-week post-treatment and found “no immediate postintervention beneficial effect of exposure to either 1.5 ATA or 2.0 ATA oxygen compared with the Sham Air intervention.” Scrutiny of the data suggests varying effects of the different doses of HBOT on the 55 outcomes, especially for four cognitive tests (California Verbal Learning Test: Long-Delay Free Recall, Index for Recognition, Short Delay Cue Recall, and Recognition Total Hits), but large standard deviations of the mean scores and separate one-way ANOVAs of the baseline and post-treatment outcomes make exact comparisons between the groups difficult. Identical to the first publication on this study no change score analysis was performed on any outcome.

The third publication (73) reported 1-week and 3-month symptom, functional, cognitive, and psychomotor outcomes and found “No significant time by intervention interaction” [*F*_(4, 63.7)_ = 1, *p* = 0.41] however, a secondary *F*-ratio test demonstrated significant improvements regardless of treatment group on five cognitive tests: Trails B, California Verbal Learning Test, Paced Auditory Serial Addition Test, Benton Visual Memory Test, and Controlled Oral Word Association Test with worsening of the Wechsler Adult Intelligence Scale-III Working Memory at 2-weeks post-treatment. Similar to the second publication (72), strict pre/post-change scores (treatment effects) for each group were not calculated, and similar to both the first (71) and second (72) publications the pre/post means of these differences were not compared between groups. Regardless, the treatment effects for each individual HBOT does/group appear small and insignificant. The net result is a suggestion of small differing effects of the three different doses of HBOT with statistically significant change only seen for reduction of PTSD symptoms in the 2 ATA oxygen group and some cognitive domains in the combined three groups.

The third comparative dosing study (81) randomized 71 military subjects 25.6 months after an average of 3.6 TBIs to receive either forty 1.2 ATA air or 1.5 ATA oxygen treatments (600 and 3120 AMs oxygen) in a 12-week period. Symptom, quality of life, neuropsychological, neurological, electroencephalography, sleep, auditory, vestibular, autonomic, visual, neuroimaging, and laboratory testing were performed before and immediately, 3 and 9 months post-treatment. Baseline characteristics suggested “worse brain injury” in the HBOT group due to significantly more frequent diffuse/traumatic axonal injury on MRIs, greater TBI symptoms (RPQ-13 and RPQ-total), PTSD symptoms (PCL-C hyperarousal score), number of combat deployments, and worse anger control. Despite this difference in injury, the compressed air group showed a deterioration in postconcussive (NSI and RPQ) and PTSD symptoms (PCL-C) during the treatment period while the HBOT group experienced significant improvement on the NSI, RPQ-3, and PCL-C compared to the compressed air group (difference in mean change score between groups: NSI: −7.6, CI = −14.4 to −0.7, *p* = 0.03; RPQ-3: −1.5, CI = −2.7 to −0.3, *p* = 0.01; RPQ-13: −5, CI = −10.7 to 0.6, *p* = 0.08; PCL-C: −7.3, CI = −13.5 to −1, *p* = 0.02), statistically moreso on the NSI and PCL-C for those subjects with PTSD. In addition, the HBOT group experienced significant improvement in some cognitive processing speed and sleep measures, and in those with PTSD improved functional balance and reduced vestibular complaints, all compared to the pressurized air treatment.

Companion publications (82, 83, 86, 88) presented components of Weaver et al. (81) in greater detail. Walker et al. (82) reported markedly disrupted sleep quality in study subjects and significant improvement in self-reports of Pittsburgh Sleep Quality Index measures (five out of eight at 13-weeks and two out of eight at 6 months) in the oxygen group compared to the air pressure group. Meehan et al. (83) found worse balance/gait measures and more affective symptoms in mTBI subjects compared to healthy controls, some within-group improvements in these domains favoring the HBOT group at 13 weeks and 6 months post-treatment, significant (but “minimal”) improvements in the HBOT group compared to the air group on balance measures, and improvements on postural control, generally favoring HBO_2_. Those with affective symptoms, particularly PTSD, who were treated with HBOT had the most improvement in postural control and otolith function. Hart et al. (86) was an extended outcome study and not pertinent to this review. Lastly, Wetzel et al. (88) demonstrated significant abnormalities in ocular metrics in the overall subject population compared to population normals. Multiple abnormalities improved in both treatment arms (pressurized air and HBOT) that persisted by 3 months post-treatment at which time the normals and study subjects were statistically indistinguishable.

In summary, the comparative dosing studies (69, 71, 81) demonstrated significant improvements in symptoms of mTBI/PPCS at 1.5 ATA oxygen and 1.3 ATA air, deterioration at 1.2 ATA air and 2.4 ATA oxygen, and no significant change at 2 ATA of pressure with 0.21, 1.5, or 2.0 ATA oxygen. In addition, significant improvements in cognition, balance, and gait measures were obtained with the 1.5 ATA oxygen dose.

The first randomized controlled trial (RCT) (70) randomized 67 civilians 33 months after a single mTBI to 40 HBOTs at 1.5 ATA oxygen or no treatment control (3,720 vs. 0 AMs oxygen) in 2 months. Mindstreams computerized testing (organized into four cognitive domains), single-photon emission computed tomography (SPECT) brain blood flow imaging, and a quality-of-life evaluation were performed pre-, within 1–3 weeks after control and treatment periods for the control crossover group, and after treatment for the HBOT group. Significant improvements were demonstrated in cognitive function and quality of life in both groups following HBOT but no significant improvement was observed following the control period. SPECT imaging revealed elevated brain activity in good agreement with the cognitive improvements. Cognitive function improvements in the HBOT group consisted of Information Processing Speed [t_(31)_ = 4.20, *p* < 0.0001], Attention [*t*_(31)_ = 3.26, *p* < 0.005], Memory [*t*_(31)_ = 4.13, *p* < 0.0005], and Executive Functions [*t*_(31)_ = 3.72, *p* < 0.0005]. Similar to the Cifu et al. studies (71–73), the outcome instrument change scores after the control period and HBOT were not statistically compared. Rather, the groups were compared at baseline and after both groups had completed HBOT. The groups were statistically indistinguishable at both comparisons and both groups had experienced significant within-in group improvements after HBOT on all instruments with medium to large effect sizes.

The second RCT (75) randomized 72 military subjects 23 months after an average of 3.1 mTBIs to one of three groups: 40 HBOTs at 1.5 ATA oxygen, 1.2 ATA air, or no HBOT (3,120, 600, or 0 AMs oxygen) during 10 weeks. Symptom questionnaires, computerized neuropsychological testing, and traditional neuropsychological testing were administered pre-, at the midpoint of treatment and after treatment for symptom questionnaires and the computerized cognitive testing, and at two time points (pre/post full treatment) for the traditional cognitive tests. There were no significant two-point change score differences between the three groups on the abbreviated RPQ (*p* = 0.24), yet a seemingly large difference between the HBOT and no-treatment group (25% of the no-treatment group, 52% of the HBOT group, and 33% of the air-pressure group); it does not appear that either treatment group was compared individually to the no-treatment group. For the total RPQ score both chamber groups demonstrated significant improvement compared to the no-treatment control group (5.4, 95% CI, −0.5 to 11.3, *P* = 0.008 HBO group; 7, 95% CI, 1.0 to 12.9, *P* = 0.02 air group) and no difference between the two-chamber groups (*P* = 0.70). Significant improvements were achieved despite many subjects not receiving the full 40 treatments (48% of the HBOT group and 59% of the “sham” group received 40 HBOTs), while symptom improvement was greater for those who completed all 40 chamber sessions. For the secondary symptom outcome score, Neurobehavioral Symptom Inventory (NSI), the no-treatment group showed a slight worsening, while both treatment groups showed improvement [Pre minus post scores: −1.1 (CI: −7.3 to 5.2, no treatment) vs. 3.7 (CI: −3.7 to 11.2, HBOT) and 6.9 (CI: 1.4 to 12.4, “sham” air). Positive scores are improvement]. Similar to the RPQ two-point change metric, it appears that neither treatment group was statistically compared to the no-treatment group. Rather, it was stated that “these change scores were not statistically different.” For PTSD symptoms, depression, generalized anxiety, pain, sleep, quality of life, and computerized cognitive testing improvements occurred in both groups, generally favoring the air treatment, but there was no statistical difference between treatment groups. Again, none of these data appear to be compared to the no-treatment control.

The third RCT (46) randomized 60 civilian and military subjects 4.6 years after an average of 3.9 mTBIs to 40 HBOTs at 1.5 ATA oxygen (3,420 AM's oxygen) in 8–9 weeks vs. no treatment (0 AM's). The no-treatment control group was then crossed over to 40 HBOTs similar to Boussi-Gross et al. (70). Subjects completed symptom and quality of life questionnaires, and neuropsychologist-administered neuropsychological, psychological, and sleep testing or questionnaires. The HBOT group experienced significant improvements compared to the control group in one of the two co-primary outcomes (NSI: mean difference in change scores between groups: −23.9 ± 9.2, CI = −29.2 to −18.6, *p* = 0.0001) with the greatest improvement in the cognitive domain. This was the largest percent improvement (52%) in symptomatic outcome for a treatment group in all of the studies reviewed. The authors noted that this outcome was likely positively biased by the daily interaction of the P.I. with the subjects during treatment (an IRB safety requirement) and completion of the symptom questionnaire at the treatment site. The 52% figure, however, was in the range of one other study outcome figure (37%) for the same symptom questionnaire and same treatment dose (75) where the P.I. and subjects were blinded to treatment. Analysis of the eight Diagnostic and Statistical Manual-IV Text Revised PPCS symptoms (2) demonstrated that the Treatment Group subjects experienced significant improvement in all eight of the PPCS definition symptoms while the Control Group experienced worsening on six of eight symptoms, similar to the Weaver et al. (81) 1.2 ATA air treatment and the Miller et al. (75) no treatment groups. The HBOT group also experienced significant improvements in Memory Index, ANAM, Hamilton Depression Scale, Hamilton Anxiety Scale, Post- Traumatic Stress Disorder Checklist, Pittsburgh Sleep Quality Index, and Quality of Life after Brain Injury compared to the Control Group. Non-significant improvement was obtained in the other co-primary outcome (Working Memory: mean difference in change scores between groups: 1.5 ± 6.5, CI = −2.23 to 5.13, *p* = 0.431. Overall, the HBOT group achieved significant improvement in 11/14 outcome instruments compared to 13/14 after HBOT in the crossed-over Control Group. Twelve of the fourteen outcome instruments were administered by the blinded neuropsychologist at the non-treatment testing site. The other two outcomes, TBI and PTSD symptom questionnaires, were collected at the treatment site by the unblinded hyperbaric technician.

### Dose Analysis of Studies

Effects of composite pressure and oxygen doses of HBOT on immediate post-treatment symptoms are presented in Table 2 for Table 1 randomized trials and case-controlled series with symptom outcomes [data abstracted from Table 7 in Harch et al. (46)]. [The Wolf et al. (69) symptom data in Table 7 was recalculated to the immediate post-treatment period using the ImPACT symptom data in Table 2 of Wolf et al. (69) and the total ImPACT score (symptoms and cognition) from Exposure Interval 6 in Figure 2 of Wolf et al. (69)]. The data show symptom improvements at 1.5 ATA oxygen and 1.3 ATA air, improvements and deteriorations at 1.2 ATA air, no effect at the normoxic oxygen control (0.21 ATA oxygen) and one dose of oxygen (1.5 ATA oxygen), both at 2 ATA pressure, an improvement at the second oxygen dose (2.0 ATA), and no change at 2.4 ATA oxygen. According to the Scorza subset analysis (113), however, and “trend toward harm” of the 2.4 ATA oxygen dose for subjects with mTBI PPCS without PTSD the actual effect of 2.4 ATA oxygen on mTBI/PPCS without PTSD is negative. The positive (75) and negative (81) outcomes at 1.2 ATA air may be explained by the 50% greater number of patients with comorbid PTSD in Miller et al. (75), a subject demographic associated with larger treatment effects in multiple studies (68, 80, 81, 87), and the 70% of subjects at risk for sleep apnea and post-treatment testing at altitude in Weaver et al. (81), phenomena that would favor negative treatment effects.

**Table 2:** Immediate post-treatment symptom changes according to pressure and oxygen dose in mTBI PPCS studies, abstracted from Table 7 in Harch et al. (46) with change in polarity.

**Pressure dose**	**No chamber treatment**	**1.2 ATA air**	**1.3 ATA air**	**1.5 ATA O_**2**_**	**2.0/.21 ATA, press/O_2_**	**2.0/1.5 ATA, press/O_2_**	**2.0/2.0 ATA, press/O_2_**	**2.4 ATA O_2_**
Oxygen dose, atmosphere-minutes	0 (46,75)	600 (75, 81)	1,002 (69)	3,120 (75, 81) 3,420 (46,80)	76 (71)	3,720 (71)	4,860 (71)	6,900 (69)
Wolf et al. (69)			+31^b*^					+2.8^b*^
Cifu et al. (71)					−1^a^	−4^a^	+12^a^	
Miller et al. (75)	+2^a^ −3^c^	+35^a^ +21^c^		+37^a^ +11^c^				
Harch et al. (80)				+36^a^				
Weaver et al. (81)		−21^a^ −13^c^		+2^a^ +10^c^				
Harch et al. (46)	+5.6^c^			+52^c^				

*Scores are percent change: Post minus Pre/Pre with polarity reversed so that positive represents symptom improvement*.

a *Rivermead Postconcussion Questionnaire*.

b *Immediate Post-Concussion Assessment and Cognitive Testing (ImPACT)*.

c *Neurobehavioral Symptom Inventory (NSI)*.

* *Values for both figures are symptom score improvements from Table 2. ImPACT symptom data in Wolf et al. (69) that were interpolated to the immediate post treatment time period, using the ImPACT total scores (symptoms plus cognition) from Figure 2 [Wolf et al. (69)] exposure Interval 6 and applying the percent reduction in composite score from Pre-exposure to Exposure Interval 6 in Figure 2 [Wolf et al. (69)] to the Pre-exposure ImPACT symptom scores in Table 2 [Wolf et al. (69)]*.

**Figure 3:**
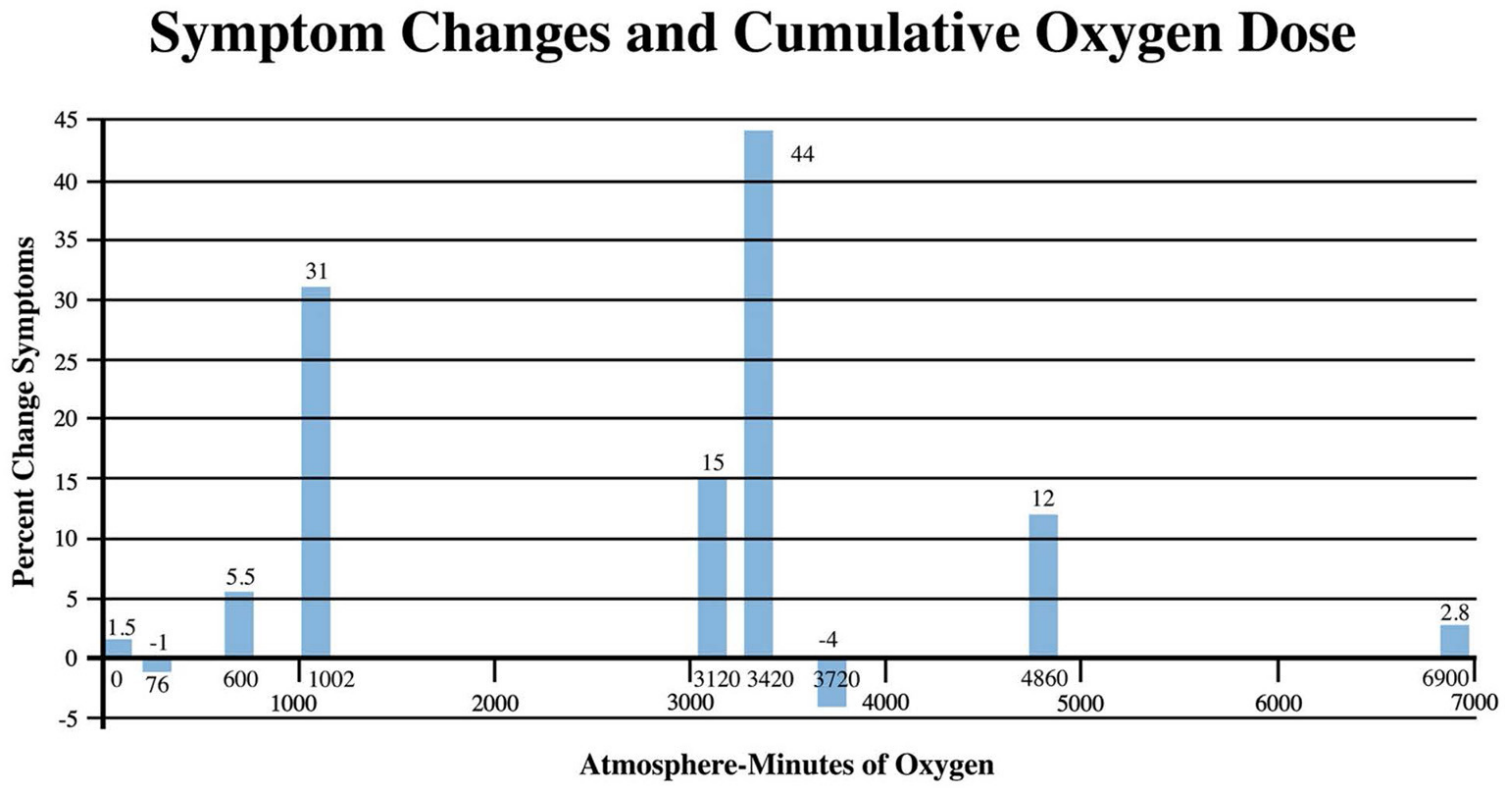
Symptom Improvements from Table 2 vs. oxygen dose for HBOT mTBI PPCS studies. Symptom percent values represent the average percent improvement in symptoms for each oxygen dose in Table 2, averaging the three different instrument (ImPACT, NSI, and RPQ) symptom outcomes.

Pressure dose effects on immediate post-treatment symptom outcomes for the studies in Table 2 are presented in Figure 2. An asymmetric bell-shaped dose-response curve shows maximum symptom improvement in the 1.3–1.5 ATA pressure range with much less improvement at lower and higher pressures. Oxygen dose effects on symptom outcomes for Table 2 studies are in Figure 3 and demonstrate a similar asymmetric bell-shaped dose-response curve with maximal results in a broad range from 1,002 to 4,860 atmosphere-minutes of oxygen and decreased responses at the lowest and highest doses of oxygen. The only aberrant value (3,720) is possibly due to the 2 ATA pressure component of the 1.5 ATA O2/2 ATA dose in the Cifu et al. study (71); the identical oxygen dose was delivered in Boussi-Gross et al. (70) at 1.5 ATA pressure with positive cognitive, mood, and functional imaging changes. Analysis of Figures 2 and 3 suggests that barometric pressure in the narrow range of 1.3–1.5 ATA has a more important effect on mTBI PPCS symptoms than oxygen pressure where a near-equal oxygen effect occurs from 1,002 to 3,420–4,860 AMs.

### Classification of Evidence, Methodologic Quality, and Risk of Bias Assessment

The CEBM Levels of Evidence (3) and American Society of Plastic Surgeons Grade Practice Recommendation grading systems (4) are presented in Tables 3, 4. The Level of Evidence and PEDro methodologic quality/bias assignments along with PEDro scoring are in Tables 5, 6. Miller et al. (75) and Harch et al. (46) meet Level 1B criteria with a minus sign to designate wide confidence intervals. Wide confidence intervals, despite significant *p*-values and moderate to large treatment effects, are likely due to TBI heterogeneity factors identified in Section Limitations (below) and small sample size. The other four randomized trials to not fit cleanly in the CEBM hierarchy. Two of the rigorously performed higher quality/low bias studies published as randomized controlled trials, Wolf et al. (69) and Cifu et al. (71), that were previously assigned Level 1 status in an mTBI/PPCS review article (100) no longer meet the definition of a randomized controlled trial due to the scientific definition of HBOT used in the present review and the recharacterization of the control groups as treatment groups. They are classified Level 2. For the same reason the Weaver et al. (81) study, a rigorously performed high quality/low bias study published as an RCT, is now a Level 2 study. The fourth of these randomized Level 2 studies, Boussi-Gross et al. (70), is a randomized and true controlled crossover trial but did not have published confidence intervals, a CEBM (3) requirement for Level 1 classification. All four of these studies are given a 2B designation with a plus sign to denote, a higher level than true Level 2 cohort studies. There is one Level 3 study (80), one Level 4 case series (84), and three Level 5 case reports (66, 67, 89). Harch et al. (68) was omitted from Table 5 due to the inclusion of its 16 subjects in Harch et al. (80).

**Table 3:** Levels of Evidence for therapeutic studies from the Centre for Evidence-Based Medicine (3) in Burns et al. (4).

**Level**	**Type of evidence**
1A	Systematic review (with homogeneity) of RCTs
1B	Individual RCT (with narrow confidence intervals)
1C	All or none study
2A	Systematic review (with homogeneity) of cohort studies
2B	Individual Cohort study (including low quality RCT, e.g., <80% follow-up)
2C	“Outcomes” research; Ecological studies
3A	Systematic review (with homogeneity) of case-control studies
3B	Individual Case-control study
4	Case series (and poor quality cohort and case-control study
5	Expert opinion without explicit critical appraisal or based on physiology bench research or “first principles”

**Table 4:** Grade practice recommendations from the American Society of Plastic Surgeons in Burns et al. (4).

**Grade**	**Descriptor**	**Qualifying evidence**	**Implications for practice**
A	Strong recommendation	Level I evidence or consistent findings from multiple studies of levels II, III, or IV	Clinicians should follow a strong recommendation unless a clear and compelling rationale for an alternative approach is present
B	Recommendation	Levels II, III, or IV evidence and findings are generally consistent	Generally, clinicians should follow a recommendation but should remain alert to new information and sensitive to patient preferences
C	Option	Levels II, III, or IV evidence, but findings are inconsistent	Clinicians should be flexible in their decision-making regarding appropriate practice, although they may set bounds on alternatives; patient preference should have a substantial influencing role
D	Option	Level V evidence: little or no systematic empirical evidence	Clinicians should consider all options in their decision making and be alert to new published evidence that clarifies the balance of benefit vs. harm; patient preference should have a substantial influencing role

*From the American Society of Plastic Surgeons Evidence-based clinical practice guidelines. Borrowed from Swanson et al. (118)*.

**Table 5:** Levels of evidence for reviewed HBOT mTBI PPCS studies and PEDro Scale methodologic quality bias ratings for randomized trials.

**Studies**	**Levels of evidence**	**Methodologic Quality/Bias analysis (PEDro Scale) (109)**
Miller et al. (75) Harch et al. (46)	1B (–) 1B (–)	9 5
Boussi-Gross et al. (70) Wolf et al. (69) Cifu et al. (71) Weaver et al. (81)	2B (+) 2B (+) 2B (+) 2B (+)	4 8 9 8
Harch et al. (80)	3B* (imaging control)	
Mozayeni (84)	4	
Harch et al. (66) Wright et al. (67) Shytle et al. (89)	5 5 5	

*Scores of: <4 are considered “poor,” 4 to 5 “fair,” 6 to 8 “good,” and 9 to 10 “excellent.”*

* *Harch et al. (68) is not included in this table due to the inclusion of its 16 subjects in Harch et al. (80)*.

**Table 6:** PEDro analysis of methodologic quality and bias for randomized trials.

**Items**	**Wolf et al. (69)**	**Boussi-Gross et al. (70)**	**Cifu et al. (71)**	**Miller et al. (75)**	**Weaver et al. (81)**	**Harch et al. (46)**
1 (eligibility criteria specified)	Y	Y	Y	Y	Y	Y
2 (random allocation)	Y	Y	Y	Y	Y	Y
3 (concealed allocation)	Y	N	Y	Y	Y	Y
4 (groups similar at baseline)	Y	Y	Y	Y	N	Y
5 (subject blinding)	Y	N	Y	Y	Y	N
6 (therapist blinding)	N	N	Y	N	N	N
7 (assessor blinding)	Y	Y	Y	Y	Y	N
8 (1 key outcome for >85% subjects)	Y	N	Y	Y	Y	N
9 (1 key outcome: intention-to-treat analysis)	N	N	N	Y	Y	N
10 (1 key outcome between group statistical comparison)	Y	N	Y	Y	Y	Y
11 (1 key outcome point measurements and variability)	Y	Y	Y	Y	Y	Y
Total:	8	4	9	9	8	5

The two randomized controlled Level 1B- trials of Miller et al. (75), and Harch et al. (46) demonstrated the benefit of the treatment groups at 1.5 ATA oxygen, and one at 1.2 ATA air, vs. control groups. The additional comparative dosing study of Weaver et al. (81) (Level 2B+) demonstrated additional proof of the efficacy of the 1.5 ATA oxygen dose compared to a 1.2 ATA air group, along with the Level 2B+randomized controlled crossover study of Boussi-Gross et al. (70), the case-controlled study of Harch et al. (80) (Level 3), the case series of Mozayeni et al. (84) (Level 4), and the 3 case reports (66, 67, 89) (Level 5).

PEDro analysis of methodological quality/bias is presented in Table 6. According to PEDro scoring statistics (119), studies scoring ≥6/10 on the PEDro scale are considered “moderate to high quality.” Using the qualitative assessment of Cashin et al. (110) for the entire scale of PEDro Scale scores, studies were rated as: poor (<4), fair (4, 5), good (6–8), and excellent (9, 10). Four (69, 71, 75, 81) of the six randomized and randomized controlled studies were good to excellent quality/low bias with PEDro scores of 8 (69), 9 (71), 9 (75), and 8 (81). Two of these four studies (75, 81) were at 1.5 ATA oxygen. The other two randomized controlled studies, both at 1.5 ATA oxygen, were fair quality/greater bias with PEDro scores of 5 (46) and 4 (70). One point of the score differential between the higher and lower quality studies is due to design differences based on the presumption by the investigators of the four higher quality/low bias studies that a low dose of pressure and oxygen were sham controls. The pseudo-shams were pressurized treatments that allowed the blinding of patients to a dose of HBOT. The two crossovers lower quality/greater bias studies (46, 70) used no-treatment control groups that prevented blinding of patients. This accounted for a loss of one point for both Boussi-Gross et al. (70) and Harch et al. (46) by PEDro analysis. In addition, the Harch et al. (46) study was scored lower on Item 7 due to the collection of one of the two primary outcomes (symptoms) at the site of treatment while the other primary outcome (working memory) and all 12 secondary outcomes were collected by the blinded neuropsychologist. Boussi-Gross et al. (70) and Harch et al. (46) also lost one point each for a significant number of dropouts after allocation: 23/90 (25%) subjects dropped out of Boussi-Gross et al. (70) after allocation due to “consent withdrawn” and an additional 12% for testing, medication, personal, and ear problems, while 6/31 subjects (19%) of the HBOT group in Harch et al. (46) dropped out (81% of allocated subjects retained) for personal, financial hardship, substance abuse, an intercurrent new diagnosis of cancer, and work conflicts (all random events) that precluded achieving the 85% allocation threshold for outcome data analysis by PEDro criteria. An additional source of bias not addressed in the PEDro analysis is bias from conflict of interest. Dr. Harch stated conflict of interest in his studies (46, 80). Many of the investigators in the low-bias studies (69, 71, 75, 81) are employed by the funding source.

### Side Effects

Side effects and adverse events were minor, but some were more frequent than expected and others were both notable and unusual; no treatment-induced serious adverse events were reported. Harch et al. (80) reported a higher incidence of minor and unusual adverse events; middle ear barotrauma (20%), exacerbation of PTSD anxiety (6.7%), and transient worsening of symptoms at the midway point (23%). Investigators attributed the high incidence of barotrauma to the twice/day frequency of HBOT in patients who developed upper respiratory infections during the treatment course. Barotrauma occurred in the prodromal phase of their upper respiratory illnesses before patients were symptomatic and could be paused from treatment. The twice/day frequency prevented the identification of those with developing infections that could have precluded barotrauma. Wolf et al. (120) documented middle ear barotrauma (5.51%) and sinus squeeze, confinement anxiety, headache, nausea, numbness, heartburn, musculoskeletal chest pain, latex allergy, and hypertension (0.07–0.61%). Boussi-Gross et al. (70) and Cifu et al. (71) did not mention/report side effects or adverse events. Mozayeni et al. (84) reported no side effects or complications. Churchill et al. (85) summarized adverse events for two DoD studies, Miller et al. (75) and Weaver et al. (81), and reported minor adverse events in 40/120 subjects (33%). Among these 40 subjects, 31 had experienced barotrauma: otic (17%), sinus (8.3%), and tooth (.8%). The high otic and sinus barotrauma figures were due to 24% otic and 11% sinus barotrauma incidences in one study (81), approximately four-fold the rates in the other study [6, 4% (75)]. Since the treatment pressures were identical in the two studies a difference in chamber operations/equipment likely accounts for the high rate and discrepancy between studies. Additional adverse events for the two studies included headache (6.7%), dizziness/vertigo (2.5%), vision change (2.5%), anxiety and somnolence (1.6% each), and dyspnea, neck irritation, eye pruritis, or hyperventilation (0.8% each). Shytle et al. (89) noted anxiety and GI discomfort in one of 3 patients at 25 HBOTs. In Harch et al. (46) middle ear barotrauma occurred in 2% along with three notable and unusual adverse events: a predicted consented perforation of a multiply previously perforated tympanic membrane in 1 patient (2%) and increasing late protocol fatigue with a transient reversal of improved cognitive PPCS symptoms in two patients at 34 and 39 HBOTs. These late deteriorations were attributed to oxidative stress.

## Discussion

Mild TBI is a heterogenous physical injury to the brain (7–27) that causes a wide range of signs, symptoms, and outcomes that have been defined in the chronic state as the Persistent PostConcussion Syndrome (2). TBI heterogeneity is reflected in the reviewed studies on HBOT in mTBI/PPCS in different subject populations (civilian and military, active duty and veteran), a wide range of ages (18–60 years), different etiologies (blunt and blast), varying times to injury [3 months (71) to 46 years (84)], PTSD or no PTSD, varied dosing protocols of pressure and oxygen, and an extensive list of outcome instruments.

HBOT is classified as a medical gas by the U.S. Centers for Drug Evaluation and Research of the FDA and consists of two components, increased barometric pressure and hyperoxia (57, 58, 112), which have been argued to have varying effects on mTBI/PPCS depending upon the doses of pressure and hyperoxia employed (46, 80, 99–101, 112). Both have demonstrated a wide range of bioactivity (56, 99, 112, 121–125), including independent, overlapping, and interactive gene expression and suppression effects (123, 124). This bioactivity is translated into wound repair and improved symptoms in diverse, mostly wound, conditions (56). This scientific understanding of hyperbaric oxygen therapy has eluded the hyperbaric medicine field for 359 years, particularly in the last 60 years, and has been confused and thwarted by the arbitrary definition of HBOT at a minimum pressure of 1.4 ATA of 100% oxygen (55, 56). To rectify the confusion, help resolve the controversy of the mTBI/PPCS studies, and enable this systemic review, the arbitrary definition was supplanted by the scientific definition (58).

Symptom outcomes were chosen as the primary outcome in this review due to their broad reflection of the heterogeneity of TBI, their applicability to medical practice, and a recommendation by the U.S. FDA to prioritize a PPCS symptom questionnaire, the NSI, as a primary outcome for an RCT on HBOT (46) in mTBI PPCS (57). This choice and the FDA's recommendation were reinforced by the results of the Weaver et al. (81) study where investigators concluded that the “NSI would be a reasonable, simple primary outcome measure in future studies” after an extensive battery of tests in all of the Department of Defense HBOT mTBI/PPCS studies revealed that “more resource-intensive measures… did not prove useful for measuring the change in this population.” The FDA also recommended that a series of clinical investigations with varying doses of pressure and hyperbaric hyperoxia should be performed (57). Inadvertently, the combination of civilian and DoD HBOT mTBI studies above have addressed these criteria with broad-based symptom outcomes (ImPACT, NSI, RPQ, or both NSI and RPQ) and a variety of pressures and levels of hyperbaric hyperoxia.

This symptom-based outcome review shows that, regardless of the heterogeneity of TBI and the studies herein, as well as negative reporting biases in studies (69, 71, 75, 81) and positive biases in studies (46, 80), analysis of the studies by outcomes, pressure dose, oxygen dose, or the composite dose of HBOT reveals that all symptom-based outcome studies performed at 1.5 ATA pressure and pressure of oxygen (46, 75, 80, 81) show symptom reductions in mTBI PPCS. Two of these 1.5 ATA HBOT studies (75, 81) are rated as CEBM Level 1 and 2 with good to excellent methodologic quality/low bias PEDro scores of 9 and 8, substantially higher than the average 5.1 scores of 37,417 PEDro scored randomized controlled physiotherapy trials (119), a category of clinical trials that share blinding challenges similar to hyperbaric medicine and surgery. The third of these 1.5 ATA oxygen trials, the RCT of Harch et al. (46) was a Level 1 study with a fair quality/greater bias PEDro Scale score of 5. The fourth study (80) is a Level 3 study. In addition, non-significant symptom reductions occurred in a single Level 2 excellent quality/low bias study at 2.0 ATA oxygen (71), and significant symptom reductions in single Level 2 and Level 1 moderate and excellent quality/low bias studies at 1.3 ATA pressure of air (69) and 1.2 ATA pressure of air (75), respectively. Effect sizes for all of the Level 1–4 studies in the present review are at least moderate and in excess of placebo effects for the oxygen groups compared to compressed air or no-treatment groups with symptom effect sizes greater than cognitive effect sizes (126).

The results achieved with the 1.5 ATA oxygen dose are reinforced when cognitive outcomes are added from these 1.5 ATA oxygen symptom outcome studies and the Boussi-Gross et al. (70) 1.5 ATA Level 2 oxygen study, the case series study of Mozayeni et al. (84), and the case reports of Wright et al. (67), and Shytle et al. (89). The symptom and cognitive outcomes are strongly supported by the functional imaging (17, 127) changes in two controlled studies (70, 80). Both studies revealed significant improvements in brain blood flow. In Harch et al. (80) the improvements in blood flow were almost exclusively in the white matter, the primary site of injury in mTBI (9, 10, 15, 22–24, 30, 32–35, 47), but significant improvements in blood flow were also demonstrated in the hippocampi consistent with the improvements in memory function of the subjects. Near identical findings, improvements in memory, and simultaneous increase in blood vessel density were demonstrated at 1.5 ATA with oxygen in the Harch et al. (65) animal model of HBOT-treated mTBI. In Boussi-Gross et al. (70) improvements in cognitive domains matched improvements in HBOT-induced brain blood flow to the anatomic areas of injury responsible for cognitive deficits seen in the subjects. In addition, the increases and decreases in brain blood flow to different regions of the brain measured in Boussi-Gross et al. (70) were an independent alternative replication of the mathematical texture analysis and visual pattern shift of brain blood flow measured in Harch et al. (80). The widespread improvements on SPECT seen in these two studies are contrary to those seen in SPECT-studied placebo drug trials (128, 129) and are consistent with the symptom and cognitive improvements seen in all of the studies at 1.5 ATA. Equally important, the beneficial outcomes with the 1.5 ATA dose were achieved with minor side effects or complications.

The outcomes achieved in the reviewed studies compare favorably with outcomes achieved in a non-controlled U.S. Department of Defense National Intrepid Center of Excellence treatment program for veterans with TBI with or without psychological health problems. DeGraba et al. (130) reported a 7.5-year experience with 1,456 veterans who were a minimum of 6 months post-TBI (average 5.09 years) and had sustained an average of 7 TBIs. Patients were treated in a 4-week intensive residential program that included over 20 different therapies, at least 20 different therapists, and multiple physicians. Results from 474 to 1,174 of the most severely symptomatic (numbers of subjects varied per outcome instrument reported) showed a decrease in NSI of 44%, Post Traumatic CheckList of 23%, General Anxiety Disorder scale-7 of 50%, and Personal Health Questionnaire-8 of 50%. Headache, a marker of TBI (131), had the least improvement (4 points, 6.5% reduction), half the amount that is considered a clinically significant change (130). The only study in this review with the same therapy benefit was the Harch et al. study (80), also a non-controlled study, whose subjects were a minimum of 6 months post-TBI (average 3.35 years) with an average of 3.5 TBIs. After 4 weeks of treatment with a single biological therapy, one technician, one physician, and a neuropsychologist subjects' RPQ decreased by 36%, Post-traumatic CheckList by 26%, General Anxiety Disorder-7 by 40%, and Personal Health Questionnaire-9 by 48%. In contrast to DeGraba et al. (130), headache, the second most responsive symptom next to dizziness (80), was reduced in 93% of subjects, indicating treatment of the underlying TBI (131). This was achieved at a cost for testing and treatment of $14,656/subject (figures from personal communication with Harch investigators adjusted from 2012 to 2021 dollars). The National Intrepid Center of Excellence is a $65 million center (132) [new centers are $14 million (133)] and the average cost of treatment in 2021 dollars adjusted from 2015 National Intrepid Center of Excellence costs were $17,153/veteran (134). U.S. Veterans Administration 2021 adjusted costs from the 2012 U.S. Congressional Budget Office report were $15,589/veteran for TBI, $18,387 for TBI with PTSD (135).

The positive findings for the 1.5 ATA oxygen dose in the mTBI PPCS studies are further supported by positive outcomes in multiple studies on HBOT at 1.5 ATA in acute severe TBI (136–143), and one study in moderate-severe subacute TBI (144), suggesting a shared sensitivity of TBI to 1.5 ATA oxygen regardless of acuity or severity. An additional RCT performed at 2.5 ATA in acute severe TBI (145) proved to be toxic, very similar to the 2.4 ATA oxygen dose in Wolf et al. (69, 113). The acute treatment course in that study (145) was interrupted in multiple subjects due to pulmonary oxygen toxicity. Similar to Wolf et al. (69), the neurological results were minimal at this high oxygen dose: no difference in mortality or duration of coma was demonstrated between groups (145). The sole positive finding was an interim outcome, increased recovery of consciousness at 30 days, in a subgroup of HBOT-treated subjects with brainstem contusion.

### Summary of Main Findings

According to the CEBM evidence classification hierarchy (3), the two randomized controlled trials of Miller et al. (75) excellent quality/low bias) and Harch et al. (46) (fair quality/greater bias) meet the Level 1 standard for Evidence-Based Medicine treatment of mTBI/PPCS. Both of these studies demonstrated the benefit of the treatment groups at 1.5 ATA oxygen, and one at 1.2 ATA air, compared to control groups. One of the studies (75) also demonstrated a positive effect on co-morbid PTSD, a finding first reported in 2009 (66) and reinforced in multiple subsequent reports (68, 69, 71, 80, 81, 87, 89). The comparative dose Level 2 studies of Boussi-Gross et al. (70) (fair quality/greater bias) and Weaver et al. (81) (good quality/low bias), the only study with mismatched treatment groups (HBOT group with more serious brain injury), demonstrated additional proof of the efficacy of the 1.5 ATA oxygen dose compared to a control group or 1.2 ATA air group, along with the case-controlled series of Harch et al. (80) (Level 3) and case series of Mozayeni et al. (84) (Levels 4), and the 3 case reports (66, 67, 89) (Level 5). At the same time, one Level 2 (good quality/low bias) study, Wolf et al. (69), demonstrated efficacy at the 1.3 ATA air dose. Compared to a previous Class B recommendation for HBOT in mTBI PPCS (100) the additional randomized controlled trials and randomized comparative dosing studies now meet CEBM Level 1 evidence and American Society of Plastic Surgeons Grade A Practice Recommendation criteria for HBOT treatment of mTBI/PPCS (4) (Tables 3–5) at 1.5 ATA oxygen.

### Limitations

The main limitation is the heterogeneity of the patients inherent in the diagnosis of mTBI/PPCS (causes of injury, circumstances, non-uniformity of force/vector/head position, anatomy, symptoms, past injuries, co-morbidities, time from injury to treatment, etc.). Additional limitations are the exclusion of non-English language literature, the discrepancy in treatment and testing sites with respect to altitude (81), small sample sizes out of proportion to the heterogeneity (46, 68–71, 75, 80, 81, 84), variability in the design of the studies (46, 68–71, 75, 80, 81, 84), heterogeneity of outcome instruments (46, 68–71, 75, 80, 81, 84), failure to appreciate that a pressure experience is not a sham such that randomized “controlled” trials were actually randomized comparative dosing studies (69, 71, 75, 81), difficulty in structuring a study with a true pressure control (46, 69–71, 75, 81), lack of statistical analysis of outcome changes of one group compared to another or control (69–71, 75), and paucity of literature to review. Despite all of these limitations which would bias data and conclusions toward rejection of the null hypothesis and Type II Error due to small sample size, the studies' data support the conclusions drawn herein.

### Conclusions

In multiple randomized and randomized controlled studies, HBOT at 1.5 ATA oxygen demonstrated statistically significant symptomatic and cognitive or cognitive improvements alone in patients with mild traumatic brain injury Persistent Postconcussion Syndrome. Positive and negative results occurred at lower and higher doses of oxygen and pressure. According to pressure and oxygen dosage analyses, increased pressure within a narrow range appears to be the more important effect than increased oxygen which is effective over a broad range. Improvements were greater when patients had comorbid Post Traumatic Stress Disorder. Large blinded controlled trials would be ideal to confirm the results of this review, but despite small sample sizes, the studies using 1.5 ATA oxygen satisfy the Centre for Evidence-Based Medicine (3) Level 1 criteria and merit a Class A Recommendation for treatment of mTBI PPCS at 1.5 ATA of oxygen, according to the American Society of Plastic Surgeons Grade Practice Recommendations (4).

## Classification of Evidence

This review provides Centre for Evidence-Based Medicine (3) Class 1 evidence that 40 hyperbaric oxygen treatments at 1.5 ATA oxygen are effective and well-tolerated in mTBI Persistent PostConcussion Syndrome. According to the American Society of Plastic Surgeons Grade Practice Recommendations, this evidence meets the threshold for and is a Grade A Practice Recommendation (4).

## Contribution to the Field Statement

Mild TBI has been traditionally considered to be innocuous. It is now appreciated to cause permanent symptoms [the Persistent PostConcussion Syndrome (PPCS)] in a substantial number of patients. Hyperbaric oxygen therapy is a treatment for mostly acute and chronic wound conditions that is scientifically defined as a dual-drug therapy that uses increased oxygen and increased pressure to treat disease pathophysiology/wounds. Multiple studies have reported confusing and seemingly conflicting results of HBOT treatment of PPCS, due to the use of a non-scientific historical definition of HBOT that led to inadvertent mal-design of some of the studies. Using the scientific definition of HBOT this review resolves the confusion and conflict by analyzing the studies on HBOT in mTBI PPCS according to individual and composite doses of pressure and oxygen. The results show that barometric pressure is the more important component of HBOT compared to the pressure of oxygen and that in multiple studies a composite dose of pressure and oxygen, 1.5 ATA oxygen, demonstrated significant improvement in symptoms and cognition in patients with PPCS. This finding has the potential to change the standard of practice in the treatment of PPCS and has substantial implications for millions of patients worldwide afflicted with PPCS, including military war veterans.

## Data Availability Statement

The original contributions presented in the study are included in the article/Supplementary Material, further inquiries can be directed to the corresponding author.

## Author Contributions

The author confirms being the sole contributor of this work and has approved it for publication.

## Funding

LSU School of Medicine paid academic protected time to partially fund the research and writing of this manuscript. The remainder of the effort was provided by the sole author without funding. The sole author paid all publication fees.

## Conflict of Interest

PH declares a co-owner of a hyperbaric service and consulting company.

## Publisher's Note

All claims expressed in this article are solely those of the authors and do not necessarily represent those of their affiliated organizations, or those of the publisher, the editors and the reviewers. Any product that may be evaluated in this article, or claim that may be made by its manufacturer, is not guaranteed or endorsed by the publisher.

## Abbreviations

AM: Atmosphere Minutes
ANAM: Automated Neuropsychological Assessment Metrics
CEBM: Centre for Evidence-Based Medicine
CNS: Central Nervous System
ImPACT: Immediate Postconcussion Assessment and Cognitive Testing
mTBI: mild Traumatic Brain Injury
NSI: Neurobehavioral Symptom Inventory
PPCS: Persistent PostConcussion Syndrome
SPECT: Single Photon Emission Computed Tomography
RPQ: Rivermead Postconcussion symptom Questionnaire.

## References

[1] Mild Traumatic Brain Injury Committee of the Head Injury Interdisciplinary Special Interest Group of the American Congress Rehabilitation Medicine. Definition of mild traumatic brain injury. J Head Trauma Rehabil. (1993) 8:86–87. 10.1097/00001199-199309000-00010

[2] Guze SB. Diagnostic and Statistical Manual of Mental Disorders, 4th ed. Washington, DC: American Psychiatric Association (2000).

[3] CEBM. Oxford Centre for Evidence-Based Medicine: Levels of Evidence (March 2009). (2009). Available online at: https://www.cebm.ox.ac.uk/resources/levels-of-evidence/oxford-centre-for-evidence-based-medicine-levels-of-evidence-march-2009 (accessed February 9, 2022).

[4] Burns PB Rohrich RJ Chung KC. The levels of evidence and their role in evidence-based medicine. Plast Reconstr Surg. (2011) 128:305–10. 10.1097/PRS.0b013e318219c171 21701348 PMC3124652

[5] Centers for Disease Control Prevention. Traumatic Brain Injury & Concussion. (2021). Available online at: https://www.cdc.gov/traumaticbraininjury/index.html (accessed September 11, 2021).

[6] American Association of Neurological Surgeons. Traumatic Brain Injury. (2021). Available online at: https://www.aans.org/Patients/Neurosurgical-Conditions-and-Treatments/Traumatic-Brain-Injury (accessed July 11, 2020).

[7] Peerless SJ Rewcastle NB. Shear injuries of the brain. Canad Med Assoc J. (1967) 96:577–82. 6020206 PMC1936040

[8] Adams JH Graham DI Murray LS Scott G. Diffuse axonal injury due to nonmissile head injury in humans: An analysis of 45 cases. Ann Neurol. (1982) 12:557–63. 10.1002/ana.410120610 7159059

[9] Povlishock JT Christman CW. The pathobiology of traumatically induced axonal injury in animals and humans: a review of current thoughts. J of Neurotrauma. (1995) 12:555–64. 10.1089/neu.1995.12.555 8683606

[10] Oppenheimer DR. Microscopic lesions in the brain following head injury. J Neurol Neurosurg Psychiat. (1968) 31:299–306. 10.1136/jnnp.31.4.299 4176429 PMC496365

[11] Symonds C. Concussion and its sequelae. Lancet. (1962) 1:1–5. 10.1016/S0140-6736(62)92635-1

[12] Cihangiroglu M Ramsey RG Dohrmann GJ. Brain injury: analysis of imaging modalities. Neurol Res. (2002) 24:7–18. 10.1179/016164102101199440 11783756

[13] Mittl RL Grossman RI Hiehle JF Hurst RW Kauder DR Gennarelli TA . Prevalence of MR evidence of diffuse axonal injury in patients with mild head injury and normal head CT findings. AJNR. (1994) 15:1583–9. 7985582 PMC8334423

[14] Voller B Benke T Benedetto K Schnider P Auff E Aichner F. Neuropsychological, MRI and EEG findings after very mild traumatic brain injury. Brain Inj. (1999) 13:821–7. 10.1080/026990599121214 10576466

[15] Wallace EJ Mathias JL Ward L. Diffusion tensor imaging changes following mild, moderate and severe adult traumatic brain injury: a meta-analysis. Brain Imaging Behav. (2018) 12:1607–21. 10.1007/s11682-018-9823-2 29383621

[16] Delano-Wood L Bangen KJ Sorg SF Clark AL Schiehser DM Luc N . Brainstem white matter integrity is related to loss of consciousness and postconcussive symptomatology in veterans with chronic mild to moderate traumatic brain injury. Brain Imaging Behav. (2015) 9:500–12. 10.1007/s11682-015-9432-2 26248618

[17] Raji CA Henderson TA. PET and single-photon emission computed tomography in brain concussion. Neuroimag Clin N Am. (2018) 28:67–82. 10.1016/j.nic.2017.09.003 29157854

[18] Kraus MF. Susmaras T Caughlin BP Walker CJ Sweeney JA Little DM. White matter integrity and cognition in chronic traumatic brain injury: a diffusion tensor imaging study. Brain. (2007) 130:2508–19. 10.1093/brain/awm216 17872928

[19] Lipton ML Gulko E Zimmerman ME Friedman BW Kim M Gellella E . Diffusion-tensor imaging implicates prefrontal axonal injury in executive function impairment following very mild traumatic brain injury. Radiology. (2009) 252:816–24. 10.1148/radiol.2523081584 19567646

[20] Mu W Catenaccio E Lipton ML. Neuroimaging in blast-related mild traumatic brain injury. J Head Trauma Rehabil. (2017) 32:55–69. 10.1097/HTR.0000000000000213 27022955

[21] Adam O Mac Donald CL Rivet D Ritter J May T Barefield M . Clinical and imaging assessment of acute combat mild traumatic brain injury in Afghanistan. Neurology. (2015) 85:219–27. 10.1212/WNL.0000000000001758 26109715 PMC4516289

[22] Shively SB Horkayne-Szakaly I Jones RV Kelly JP Armstrong RC Perl DP. Characterisation of interface astroglial scarring in the human brain after blast exposure: a post-mortem case series. Lancet Neurol. (2016) 15:944–53. 10.1016/S1474-4422(16)30057-6 27291520

[23] Povlishock JT. Traumatically induced axonal injury: pathogenesis and pathobiological implications. Brain Pathol. (1992) 2:1–12. 1341941

[24] Ryu J Horkayne-Szakaly I Xu L Pletnikova O Leri F Eberhart C . The problem of axonal injury in the brains of veterans with histories of blast exposure. Acta Neuropathol Commun. (2014) 2:153. 10.1186/s40478-014-0153-3 25422066 PMC4260204

[25] Evans RW. The postconcussion syndrome and the sequelae of mild head injury. Neurol Clin. (1992) 10:815–47. 10.1016/S0733-8619(18)30182-8 1435659

[26] MacKenzie JD Siddiqi F Babb JS Bagley LJ Mannon LJ Sinson GP . Brain atrophy in mild or moderate traumatic brain injury: a longitudinal quantitative analysis. AJNR Am J Neuroradiol. (2002) 23:1509–15. 12372740 PMC7976797

[27] Hofman PA Stapert SZ van Kroonenburgh MJ Jolles J de Kruijk J Wilmink JT. MR imaging, single-photon emission CT, and neurocognitive performance after mild traumatic brain injury. AJNR Am J Neuroradiol. (2001) 22:441–9. 11237964 PMC7976823

[28] Strich SJ Oxon DM. Shearing of nerve fibres as a cause of brain damage due to head injury: a pathological study of twenty cases. Lancet. (1961) 1961:443–8. 10.1016/S0140-6736(61)92426-6

[29] Adams JH Graham DI Scott G Parker LS Doyle D. Brain damage in fatal non-missile head injury. J Clin Pathol. (1980) 33:1132–45. 10.1136/jcp.33.12.1132 7451661 PMC1146364

[30] Pearn ML Niesman IR Egawa J Sawada A Almenar-Queralt A Shah SB . Pathophysiology associated with traumatic brain injury: current treatments and potential novel therapeutics. Cell Mol Neurobiol. (2017) 37:571–85. 10.1007/s10571-016-0400-1 27383839 PMC11482200

[31] Roth TL Nayak D Atanasijevic T Koretsky AP Latour LL McGavern DB. Transcranial amelioration of inflammation and cell death after brain injury. Nature. (2014) 505:223–35. 10.1038/nature12808 24317693 PMC3930079

[32] Povlishock JT Becker DP Cheng CLY Vaughan GW. Axonal change in minor head injury. J Neuropath Exper Neurol. (1983) 42:225–42. 10.1097/00005072-198305000-00002 6188807

[33] Kimura H Meaney DF McGowan JC Grossman RI Lenkinski RE Ross DT . Magnetization transfer imaging of diffuse axonal injury following experimental brain injury in the pig: characterization by magnetization transfer ratio with histopathologic correlation. J Comput Assist Tomogr. (1996) 20:540–6. 10.1097/00004728-199607000-00007 8708052

[34] Bauman RA Ling G Tong L Januszkiewicz A Agoston D Delanerolle N . An introductory characterization of a combat-casualty-care relevant swine model of closed head injury resulting from exposure to explosive blast. J Neurotrauma. (2009) 26:841–60. 10.1089/neu.2008.0898 19215189

[35] Agamanolis DP. Chapter 4, Traumatic brain injury and increased intracranial pressure. In: Neuropathology, an Illustrated Interactive Course for Medical Students and Residents. Rootstown, OH: Northeast Ohio Medical University (2017). Available online at: https://neuropathology-web.org/chapter4/chapter4bContusions_dai_sbs.html (accessed February 9, 2022).

[36] Bouma GJ Muizelaar JP Choi SC Newton PG Young HF. Cerebral circulation and metabolism after severe traumatic brain injury: the elusive role of ischemia. J Neurosurg. (1991) 75:685–93. 10.3171/jns.1991.75.5.0685 1919689

[37] van den Brink WA van Santbrink H Avezaat CJ Hogesteeger C Jansen W Kloos LM . Monitoring brain oxygen tension in severe head injury: the Rotterdam experience. Acta Neurochir Suppl. (1998) 71:190–4. 10.1007/978-3-7091-6475-4_55 9779181

[38] Zhi DS Zhang S Zhou LG. Continuous monitoring of brain tissue oxygen pressure in patients with severe head injury during moderate hypothermia. Surg Neurol. (1999) 52:393–6. 10.1016/S0090-3019(99)00101-9 10555846

[39] Schoettle RJ Kochanek PM Magargee MJ Uhl MW Nemoto EM. Early polymorphonuclear leukocyte accumulation correlates with the development of posttraumatic cerebral edema in rats. J Neurotrauma. (1990) 7:207–17. 10.1089/neu.1990.7.207 2127947

[40] Bullock R Smith R Favier J Trevou M du Blake G. Brain specific gravity and CT scan density measurements after human head injury. J Neurosurg. (1985) 63:64–8. 10.3171/jns.1985.63.1.0064 4009276

[41] Martin NA Doberstein C Alexander M Khanna R Benalcazar H Alsina G . Posttraumatic cerebral arterial spasm. J Neurotrauma. (1995) 12:897–901. 10.1089/neu.1995.12.897 8594217

[42] Zurynski YA Dorsch NWC. A review of cerebral vasospasm. Part IV. Post-traumatic vasospasm. J Clin Neurosci. (1998) 5:146–54. 10.1016/S0967-5868(98)90028-5 18639002

[43] McIntosh TK Smith DH Garde E. Therapeutic approaches for the prevention of secondary brain injury. Euro J Anaesthesiol. (1996) 13:291–309. 10.1097/00003643-199605000-00007 8737120

[44] Hovda DA Lee SM Smith ML Von Stuck S Bergsneider M Kelly D . The neurochemical and metabolic cascade following brain injury: moving from animal models to man. J Neurotrauma. (1995) 12:903–6. 10.1089/neu.1995.12.903 8594218

[45] Zhuang J Shackford SR Schmoker JD Anderson ML. The association of leukocytes with secondary brain injury. J Trauma. (1993) 35:415–422. 10.1097/00005373-199309000-00014 8371301

[46] Harch PG Andrews SR Rowe CJ Lischka JR Townsend MH Yu Q . Hyperbaric oxygen therapy for mild traumatic brain injury persistent postconcussion syndrome: a randomized controlled trial. Med Gas Res. (2020) 10:8–20. 10.4103/2045-9912.279978 32189664 PMC7871939

[47] Jane JA Steward O Gennarelli T. Axonal degeneration induced by experimental noninvasive minor head injury. J Neurosurg. (1985) 62:96–100. 10.3171/jns.1985.62.1.0096 3964861

[48] Smith DH Meaney DF Lenkinski RE Alsop DC Grossman R Kimura H . New magnetic resonance imaging techniques for the evaluation of traumatic brain injury. J Neurotrauma. (1995) 12:573–7. 10.1089/neu.1995.12.573 8683608

[49] McInnes K Friesen CL MacKenzie DE Westwood DA Boe SG. Mild traumatic brain injury (mTBI) and chronic cognitive impairment: a scoping review. PLoS ONE. (2017) 12:e0174847. 10.1371/journal.pone.0174847 28399158 PMC5388340

[50] Hiploylee C Dufort PA Davis HS Wennberg RA Tartaglia MC Mikulis D . Longitudinal study of postconcussion syndrome: not everyone recovers. J Neurotrauma. (2017) 34:1511–23. 10.1089/neu.2016.4677 27784191 PMC5397249

[51] Congress Congress of the United States Congressional Budget, Office. A CBO Study: The Veterans Health Administration's Treatment of PTSD and Traumatic Brain Injury Among Recent Combat Veterans. (2012). Available online at: https://www.cbo.gov/sites/default/files/cbofiles/attachments/02-09-PTSD.pdf (accessed September 11, 2021).

[52] Cooper DB Bunner AE Kennedy JE Balldin V Tate DF Eapen BC . Treatment of persistent post-concussive symptoms after mild traumatic brain injury: a systematic review of cognitive rehabilitation and behavioral health interventions in military service members and veterans. Brain Imag Behav. (2015) 9:403–20. 10.1007/s11682-015-9440-2 26330376

[53] Diaz-Arrastia R Kochanek PM Bergold P Kenney K Marx CE Grimes JB . Pharmacotherapy of traumatic brain injury: state of the science and the road forward: report of the department of defense neurotrauma pharmacology workgroup. J Neurotrauma. (2014) 31:135–58. 10.1089/neu.2013.3019 23968241 PMC3900003

[54] Wilson SH Roth M Lindblad AS Weaver LK. Review of recent non-hyperbaric oxygen interventions for mild traumatic brain injury. Undersea Hyperb Med. (2016) 43:615–27. 28768077

[55] Hampson NB (editor). Hyperbaric Oxygen Therapy: 1999 Committee Report. Kensington, MD: Undersea and Hyperbaric Medical Society (1999).

[56] Moon RE (editor). Hyperbaric Oxygen Therapy Indications, 14th ed. North Palm Beach, FL: Best Publishing Company (2019).

[57] Harch PG. Oxygen and pressure epigenetics: understanding hyperbaric oxygen therapy after 355 years as the oldest gene therapy known to man. Townsend Lett. (2018) 417:30–4. Available online at: https://www.townsendletter.com/article/oxygen-and-pressure-epigenetics-understanding-hyperbaric-oxygen-therapy-after-355-years-as-the-oldest-gene-therapy-known-to-man/

[58] Harch PG. HBO therapy in global cerebral ischemia/anoxia and coma. In: Jain KK editor. Textbook of Hyperbaric Medicine, 6th ed. Cham: Springer (2017). p. 269–319. 10.1007/978-3-319-47140-2_20

[59] Centers for Medicare Medicaid Services. National Coverage Determination (NCD) Hyperbaric Oxygen Therapy (20.29). National Coverage Determinations (NCD) Manual (2017). Available online at: https://www.cms.gov/medicare-coverage-database/details/ncd-details.aspx?ncdid=12&ver=3 (accessed September 11, 2021). 26110197

[60] Jain KK. Worldwide Overview of Hyperbaric Medicine. In: Jain KK editor. Textbook of Hyperbaric Medicine, 6th ed. Cham: Springer (2017). p. 609–14. 10.1007/978-3-319-47140-2_49

[61] Takahashi H Yagi H. Hyperbaric Medicine in Japan. In: Jain KK editor. Textbook of Hyperbaric Medicine, 5th ed. Gottingen: Hogrefe and Huber Publishers (2009). p. 495–8.

[62] Mathieu D Marroni A Kot J. Consensus Conference: Tenth European Consensus Conference on Hyperbaric Medicine: recommendations for accepted and non-accepted clinical indications and practice of hyperbaric oxygen treatment. Diving Hyperb Med. (2017) 47:24–32. 10.28920/dhm47.2.131-132 28357821 PMC6147240

[63] Harch PG. Hyperbaric oxygen therapy in the treatment of chronic traumatic brain injury: from Louisiana boxers to U.S. veterans. Wound Care Hyperb Med. (2010) 1:26–34.

[64] Harch PG Van Meter KW Gottlieb SF Staab P. HMPAO SPECT brain imaging and low pressure HBOT in the diagnosis and treatment of chronic traumatic, ischemic, hypoxic, and anoxic encephalopathies. Undersea Hyperb Med. (1994) 21(Suppl):30.

[65] Harch PG Kriedt C Van Meter KW Sutherland RJ. Hyperbaric oxygen therapy improves spatial learning and memory in a rat model of chronic traumatic brain injury. Brain Res. (2007) 1174:120–9. 10.1016/j.brainres.2007.06.105 17869230

[66] Harch PG Fogarty EF Staab PK Van Meter K. Low pressure hyperbaric oxygen therapy and SPECT brain imaging in the treatment of blast-induced chronic traumatic brain injury (post-concussion syndrome) and post-traumatic stress disorder: a case report. Cases J. (2009) 2:6538. 10.4076/1757-1626-2-6538 19829822 PMC2740054

[67] Wright JK Zant E Groom K Schlegel RE Gilliland K. Case report: treatment of mild traumatic brain injury with hyperbaric oxygen. Undersea Hyperb Med. (2009) 36:391–9. 20112530

[68] Harch PG Andrews SR Fogarty EF Amen D Pezzullo JC Lucarini J . A phase I study of low-pressure hyperbaric oxygen therapy for blast-induced post-concussion syndrome and post-traumatic stress disorder. J Neurotrauma. (2012) 29:168–85. 10.1089/neu.2011.1895 22026588

[69] Wolf G Cifu DX Baugh L Carne W Profenna L. The effect of hyperbaric oxygen on symptoms following mild traumatic brain injury. J Neurotrauma. (2012) 29:2606–12. 10.1089/neu.2012.2549 23031217

[70] Boussi-Gross R Golan H Fishlev G Bechor Y Volkov O Bergan J . Hyperbaric oxygen therapy can improve post concussion syndrome years after mild traumatic brain injury-randomized prospective trial. PLoS ONE. (2013) 8:e0079995. 10.1371/journal.pone.0079995 24260334 PMC3829860

[71] Cifu DX Hart BB West SL Walker W Carne W. The effect of hyperbaric oxygen on persistent postconcussion symptoms. J Head Trauma Rehabil. (2014) 29:11–20. 10.1097/HTR.0b013e3182a6aaf0 24052094

[72] Walker WC Franke LM Cifu DX Hart BB. Randomized, sham-controlled, feasibility trial of hyperbaric oxygen for service members with postconcussion syndrome: cognitive and psychomotor outcomes one week postintervention. Neurorehabil Neural Repair. (2014) 28:420–32. 10.1177/1545968313516869 24370568

[73] Cifu DX Walker WC West SL Hart BB Franke LM Sima A . Hyperbaric oxygen for blast-related post-concussion syndrome: three-month outcomes. Ann Neurol. (2014) 75:277–86. 10.1002/ana.24067 24255008

[74] Cifu DX Hoke KW Wetzel PA Wares JR Gitchel G Carne W. Effects of hyperbaric oxygen on eye tracking abnormalities in males after mild traumatic brain injury. J Rehabil Res Dev. (2014) 51:1047–56. 10.1682/JRRD.2014.01.0013 25436771

[75] Miller RS Weaver LK Bahraini N Churchill S Price RC Skiba V . Effects of hyperbaric oxygen on symptoms and quality of life among service members with persistent postconcussion symptoms: a randomized clinical trial. JAMA Intern Med. (2015) 175:43–52. 10.1001/jamainternmed.2014.5479 25401463

[76] Wolf EG Baugh LM Schubert-Kabban CM Richards MF Prye J. Cognitive function in a traumatic brain injury hyperbaric oxygen randomized trial. Undersea Hyperb Med. (2015) 42:313–32. 26403017

[77] Churchill S Miller RS Deru K Wilson SH Weaver LK. Simple and procedural reaction time for mild traumatic brain injury in a hyperbaric oxygen clinical trial. Milit Med. (2016) 181:40–4. 10.7205/MILMED-D-15-00148 27168551

[78] Skipper LD Churchill S Wilson SH Deru K Labutta RJ Hart BB. Hyperbaric oxygen for persistent post-concussive symptoms: long-term follow-up. Undersea Hyperb Med. (2016) 43:601–13. 28768076

[79] Shandley S Wolf EG Schubert-Kabban CM Baugh LM Richards MF Prye J . Increased circulating stem cells and better cognitive performance in traumatic brain injury subjects following hyperbaric oxygen therapy. Undersea Hyperb Med. (2017) 44:257–69. 10.22462/5.6.2017.6 28779582

[80] Harch PG Andrews SR Fogarty EF Lucarini J Van Meter KW. Case control study: hyperbaric oxygen treatment of mild traumatic brain injury persistent post-concussion syndrome and post-traumatic stress disorder. Med Gas Res. (2017) 7:156–74. 10.4103/2045-9912.215745 29152209 PMC5674654

[81] Weaver LK Wilson SH Lindblad AS Churchill S Deru K Price RC . Hyperbaric oxygen for post-concussive symptoms in United States military service members: a randomized clinical trial. Undersea Hyperb Med. (2018) 45:129–56. 29734566

[82] Walker JM Mulatya C Hebert D Wilson SH Lindblad AS Weaver LK. Sleep assessment in a randomized trial of hyperbaric oxygen in US service members with post concussive mild traumatic brain injury compared to normal controls. Sleep Med. (2018) 51:66–79. 10.1016/j.sleep.2018.06.006 30099354

[83] Meehan A Hebert D Deru K Weaver LK. Longitudinal study of hyperbaric oxygen intervention on balance and affective symptoms in military service members with persistent post-concussive symptoms. J Vestib Res. (2019) 29:205–19. 10.3233/VES-180671 31282447

[84] Mozayeni BR Duncan W Zant E Love TL Beckman RL Stoller KP. The National Brain Injury, Rescue and Rehabiliation Study-a multicenter observational study of hyperbaric oxygen for mild traumatic brain injury with post-concussive symptoms. Med Gas Res. (2019) 9:1–12. 10.4103/2045-9912.254636 30950414 PMC6463441

[85] Churchill S Deru K Weaver LK Wilson SH Hebert D Miller RS . Adverse events and blinding in two randomized trials of hyperbaric oxygen for persistent post-concussive symptoms. Undersea Hyperb Med. (2019) 46:331–40. 10.22462/13.15.2019.10 31394602

[86] Hart BB Wilson SH Churchill S Deru K Weaver LK Minnakanti M . Extended follow-up in a randomized trial of hyperbaric oxygen for persistent post-concussive symptons. Undersea Hyperb Med. (2019) 46:313–27. 10.22462/13.15.2019.9 31394601

[87] Hart BB Weaver LK Gupta A Wilson SH Vijayarangan A Deru K . Hyperbaric oxygen for mTBI-associated PCS and PTSD: Pooled analysis of results from Department of Defense and other published studies. Undersea Hyperb Med. (2019) 46:353–83. 10.22462/13.15.2019.12 31394604

[88] Wetzel PA Lindblad AS Mulatya C Kannan MA Villmar Z Gitchel GT . Eye tracker outcomes in a randomized trial of 40 sessions of hyperbaric oxygen or sham in participants with persistent post concussive symptoms. Undersea Hyperb Med. (2019) 46:299–311. 10.22462/13.15.2019.8 31394600

[89] Shytle RD Eve DJ Kim S-H Spiegel A Sanberg PR Borlongan CV. Retrospective case series of traumatic brain injury and post-traumatic stress disorder treated with hyperbaric oxygen therapy. Cell Transplant. (2019) 28:885–92. 10.1177/0963689719853232 31134828 PMC6719491

[90] Harch PG Van Meter KW Neubauer RA Gottlieb SF. Use of HMPAO SPECT for assessment of response to HBO in ischemic/hypoxic encephalopathies. In: Jain KK editor. Textbook of Hyperbaric Medicine, 2nd ed. Seattle, WA: Hogrefe and Huber Publishers (1996). p. 480–91.

[91] Harch PG Neubauer RA. Hyperbaric oxygen therapy in global cerebral ischemia/anoxia and coma. In: Jain KK editor. Textbook of Hyperbaric Medicine, 3rd Revised Edition. Seattle, WA: Hogrefe and Huber Publishers (1999). p. 319–45.

[92] Harch PG Neubauer RA. Hyperbaric oxygen therapy in global cerebral ischemia/anoxia and coma. In: Jain KK editor. Textbook of Hyperbaric Medicine, 3rd Revised Edition. Seattle, WA: Hogrefe and Huber Publishers (2004). p. 223–61.

[93] Harch PG Neubauer RA. Hyperbaric oxygen therapy in global cerebral ischemia/anoxia and coma.” In: Jain KK editor. Textbook of Hyperbaric Medicine, 5th Revised Edition. Seattle, WA: Hogrefe and Huber Publishers (2009). p. 235–74.

[94] Harch PG Neubauer RA Uszler JM James PB. Diagnostic imaging and hbo therapy. In: Jain KK editor. Textbook of Hyperbaric Medicine, 5th Revised Edition. Seattle, WA: Hogrefe and Huber Publishers (2009). p. 505–19.

[95] Neubauer RA Gottlieb SF Pevsner NH. Hyperbaric oxygen for treatment of closed head injury. South Med J. (1994) 87:933–6. 10.1097/00007611-199409000-00015 8091261

[96] Golden ZL Neubauer R Golden CJ Greene L Marsh J Mleko A. Improvement in cerebral metabolism in chronic brain injury after hyperbaric oxygen therapy. Int J Neurosci. (2002) 112:119–31. 10.1080/00207450212027 12325401

[97] Golden Z Golden CJ Neubauer RA. Improving neuropsychological function after chronic brain injury with hyperbaric oxygen. Disabil Rehabil. (2006) 28:1379–86. 10.1080/09638280600638364 17071569

[98] Churchill S Weaver LK Deru K Russo AA Handrahan D Orrison WW Jr . A prospective trial of hyperbaric oxygen for chronic sequelae after brain injury (HYBOBI). Undersea Hyperb Med. (2013) 40:165–93. 23682548

[99] Harch PG. Hyperbaric oxygen therapy for post-concussion syndrome: contradictory conclusions from a study mischaracterized as sham-controlled. J Neurotrauma. (2013) 30:1995–9. 10.1089/neu.2012.2799 24004322 PMC3837504

[100] Figueroa XA Wright JK. Hyperbaric oxygen: B-level evidence in mild traumatic brain injury clinical trials. Neurology. (2016) 87:1–7. 10.1212/WNL.0000000000003146 27581219

[101] Hadanny A Efrati S. Treatment of persistent post-concussion syndrome due to mild traumatic brain injury: current status and future directions. Expert Rev Neurother. (2016) 16:875–87. 10.1080/14737175.2016.1205487 27337294

[102] Marois P Mukherjee A Ballaz L. Hyperbaric oxygen treatment for persistent postconcussion symptoms—a placebo effect? JAMA Intern Med. (2015) 175:1239–40. 10.1001/jamainternmed.2015.1032 26146912

[103] Mychaskiw G Stephens PL. Hyperbaric oxygen, mild traumatic brain injury, and study design: an elusive target. J Neurotrauma. (2013) 30:1681–2. 10.1089/neu.2012.2805 23270538

[104] Mychaskiw G. Known knowns, known unknowns and unknown unknowns: the science and the passion of HBO2 therapy and traumatic brain injury: an editorial perspective. Undersea Hyperb Med. (2013) 40:371–2. 24224278

[105] Hu Q Manaenko A Guo Z Huang L Tang J Zhang JH. Hyperbaric oxygen therapy for post concussion symptoms: issues may affect the results. Med Gas Res. (2015) 5:10. 10.1186/s13618-015-0033-3 26306183 PMC4547434

[106] Weaver LK Cifu D Hart B Wolf G Miller S. Hyperbaric oxygen for post-concussion syndrome: design of Department of Defense clinical trials. Undersea Hyperb Med. (2012) 39:807–14. 22908837

[107] Mitchell SJ Bennett MH. Unestablished indications for hyperbaric oxygen therapy. Diving Hyperb Med. (2014) 44:228–34. 25596836

[108] Page MJ McKenzie JE Bossuyt PM Boutron I Hoffmann TC Mulrow CD . The PRISMA 2020 statement: an updated guideline for reporting systematic reviews. BMJ. (2021) 372:n71. 10.1136/bmj.n71 33782057 PMC8005924

[109] Maher CG Sherrington C Herbert RD Moseley AM Elkins M. Reliability of the PEDro scale for rating quality of randomized controlled trials. Phys Ther. (2003) 83:713–21. 10.1093/ptj/83.8.713 12882612

[110] Cashin AG McAuley JH. Clinimetrics: Physiotherapy Evidence Database (PEDro) scale. J Physiotherapy. (2020) 66:59. 10.1016/j.jphys.2019.08.005 31521549

[111] Ma J Hong G Ha E Hong H Kim J Joo Y . Hippocampal cerebral blood flow increased following low-pressure hyperbaric oxygenation in firefighters with mild traumatic brain injury and emotional distress. Neurol Sci. (2021) 42:4131–8. 10.1007/s10072-021-05094-5 33532950

[112] Harch PG. Hyperbaric oxygen in chronic traumatic brain injury: oxygen, pressure, and gene therapy. Med Gas Res. (2015) 5:9. 10.1186/s13618-015-0030-6 26171141 PMC4499900

[113] Scorza KA McCarthy W Miller RS Carne W Wolf GW. Hyperbaric oxygen effects on PTSD and mTBI symptoms: a subset analysis. In: Undersea and Hyperbaric Medical Society Annual Meeting. Orlando, FL. (2013).

[114] Heyboer M Milovanova TN Wojcik S Grant W Chin M Hardy KR . CD34+/CD45-dim stem cell mobilization by hyperbaric oxygen-changes with oxygen dose. Stem Cell Res. (2014) 12:638–45. 10.1016/j.scr.2014.02.005 24642336 PMC4037447

[115] Lee YS Chio CC Chang CP Wang LC Chiang PM Niu KC . Long course hyperbaric oxygen stimulates neurogenesis and attenuates inflammation after ischemic stroke. Mediators Inflamm. (2013) 2013:512978. 10.1155/2013/512978 23533308 PMC3595722

[116] Yang Y Wei H Zhou X Zhang F Wang C. Hyperbaric oxygen promotes neural stem cell proliferation by activating vascular endothelial growth factor/extracellular signal-regulated kinase signaling after traumatic brain injury. Neuroreport. (2017) 28:1232–8. 10.1097/WNR.0000000000000901 28953090

[117] Zhang T Yang QW Wang SN Wang JZ Wang Q Wang Y . Hyperbaric oxygen therapy improves neurogenesis and brain blood supply in piriform cortex in rats with vascular dementia. Brain Injury. (2010) 24:1350–7. 10.3109/02699052.2010.504525 20715898

[118] Swanson JA Schmitz D Chung KC. How to practice evidence-based medicine. Plastic Reconstr Surg. (2010) 126:286–94. 10.1097/PRS.0b013e3181dc54ee 20224459 PMC4389891

[119] PEDro Statistics. Available online at: https://pedro.org.au/english/learn/pedro-statistics/ (accessed February 9, 2022).

[120] Wolf EG Prye J Michaelson R Brower G Profenna L Boneta O. Hyperbaric side effects in a traumatic brain injury randomized trial. Undersea Hyperb Med. (2012) 39:1075–82. 23342764

[121] Macdonald AG Fraser PJ. The transduction of very small hydrostatic pressures. Comp Biochem Physiol A Mol Integr Physiol. (1999) 122:13–36. 10.1016/S1095-6433(98)10173-3 10216930

[122] Oh S Lee E Lee J Lim Y Kim J Woo S. Comparison of the effects of 40% oxygen and two atmospheric absolute air pressure conditions on stress-induced premature senescence of normal human diploid fibroblasts. Cell Stress Chaperones. (2008) 13:447–58. 10.1007/s12192-008-0041-5 18465208 PMC2673923

[123] Chen Y Nadi NS Chavko M Auker CR McCarron RM. Microarray analysis of gene expression in rat cortical neurons exposed to hyperbaric air and oxygen. Neurochem Res. (2009) 34:1047–56. 10.1007/s11064-008-9873-8 19015983

[124] Godman CA Chheda KP Hightower LE Perdrizet G Shin D-G Giardina C. Hyperbaric oxygen induces a cytoprotective and angiogenic response in human microvascular endothelial cells. Cell Stress Chaperones. (2010) 15:431–42. 10.1007/s12192-009-0159-0 19949909 PMC3082642

[125] Kendall AC Whatmore JL Harries LW Winyard PG Eggleton P Smerdon GR. Different oxygen treatment pressures alter inflammatory gene expression in human endothelial cells. Undersea Hyperb Med. (2013) 40:115–23. 23682543

[126] Biggs AT Dainer HM Littlejohn LF. Effect sizes for symptomatic and cognitive improvements in traumatic brain injury following hyperbaric oxygen therapy. J Appl Physiol. (1985) (2021) 130:1594–603. 10.1152/japplphysiol.01084.2020 33792399 PMC8354823

[127] Raji CA Tarzwell R Pavel D Schneider H Uszler M Thornton J . Clinical utility of SPECT neuroimaging in the diagnosis and treatment of traumatic braininjury: a systematic review. PLoS ONE. (2014) 9:e91088. 10.1371/journal.pone.0091088 24646878 PMC3960124

[128] Beauregard M. Effect of mind on brain activity: evidence from neuroimaging studies of psychotherapy and placebo effect. Nord J Psychiatry. (2009) 63:5–16. 10.1080/08039480802421182 19023697

[129] Jarcho JM Mayer EA London ED. Neuroimaging placebo effects: new tools generate new questions. Clin Pharmacol Ther. (2009) 86:352–4. 10.1038/clpt.2009.126 19763112 PMC3345504

[130] DeGraba TJ Williams K Koffman R Bell JL Pettit W Kelly JP . Efficacy of an interdisciplinary intensive outpatient program in treating combat-related traumatic brain injury and psychological health conditions. Front Neurol. (2020) 11:580182. 10.3389/fneur.2020.580182 33536993 PMC7848806

[131] Hoge CW McGurk D Thomas JL Cox AL Engel CC Castro CA. Mild traumatic brain injury in US soldiers returning from Iraq. N Engl J Med. (2008) 358:453–63. 10.1056/NEJMoa072972 18234750

[132] JSTOR. History and Mission of the NICoE. (2015). Available online at: https://www.jstor.org/stable/10.7249/j.ctt15sk8fw.11?seq=1#metadata_info_tab_contents (accessed September 11, 2021).

[133] Intrepid Spirit Centers. Intrepid Fallen Heroes Fund. (2021). Available online at: https://www.fallenheroesfund.org/intrepid-spirit (accessed February 9, 2022).

[134] Biospace. NIH's Report “New (TBI) Treatments are Needed” Confirms Time for “Say Goodbye TBI Campaign”. (2017). Available online at: https://www.biospace.com/article/releases/nih-s-report-new-tbi-treatments-are-needed-confirms-time-for-say-goodbye-tbi-campaign-/?keywords=Dr.+Theodore+Henderson (accessed September 11, 2021).

[135] Congressional Budget Office. Treatment of PTSD Traumatic Brain Injury by the Veterans Health Administration. (2012). Available online at: https://www.cbo.gov/publication/42980 (accessed February 9, 2022).

[136] Holbach KH Caroli A Wassmann H. Cerebral energy metabolism in patients with brain lesions at normo- and hyperbaric oxygen pressures. J Neurol. (1977) 217:17–30. 10.1007/BF00316313 75249

[137] Holbach KH Wassman H Caroli A. Continuous rCBF measurements during hyperbaric oxygenation. In: JSmith G editor. Proceeedings of the 6th International Congress on Hyperbaric Medicine. Aberdeen: Aberdeen University Press (1977). p. 104–11.

[138] Holbach KH Wassman H Caroli A. Correlation between electroencephalographical and rCBF changes during hyperbaric oxygenation. In: JSmith G editor. Proceedings of the 6th International Congress on Hyperbaric Medicine. Aberdeen: Aberdeen University Press (1977). p. 112–7.

[139] Holbach KH Wassmann H Kolberg T. Verbesserte Reversibilität des Traumatischen Mittelhirnsyndromes bei Anwendung der Hyperbaren Oxygenierung. (Improved reversibility of the traumatic midbrain syndrome following the use of hyperbaric oxygenation. Acta Neurochir. (1974) 30:247–56. 10.1007/BF01405583 4432786

[140] Rockswold GL Ford SE Anderson DC Bergman TA Sherman RF. Results of a prospective randomized trial for treatment of severely brain-injured patients with hyperbaric oxygen. J Neurosurg. (1992) 76:929–34. 10.3171/jns.1992.76.6.0929 1588426

[141] Rockswold SB Rockswold GL Vargo JM Erickson CA Sutton RL Bergman TA . Effects of hyperbaric oxygenation therapy on cerebral metabolism and intracranial pressure in severely brain injured patients. J Neurosurg. (2001) 94:403–11. 10.3171/jns.2001.94.3.0403 11235943

[142] Rockswold SB Rockswold GL Zaun DA Zhang X Cerra CE Bergman TA . A prospective, randomized clinical trial to compare the effect of hyperbaric to normobaric hyperoxia on cerebral metabolism, intracranial pressure, and oxygen toxicity in severe traumatic brain injury. J Neurosurg. (2010) 112:1080–94. 10.3171/2009.7.JNS09363 19852540

[143] Rockswold SB Rockswold GL Zaun DA Liu J. A prospective, randomized Phase II clinical trial to evaluate the effect of combined hyperbaric and normobaric hyperoxia on cerebral metabolism, intracranial pressure, oxygen toxicity, and clinical outcome in severe traumatic brain injury. J Neurosurg. (2013) 118:1317–28. 10.3171/2013.2.JNS121468 23510092 PMC12928550

[144] Sahni T Jain M Prasad R Sogani SK Singh VP. Use of hyperbaric oxygen in traumatic brain injury: retrospective analysis of data of 20 patients treated at a tertiary care centre. British J Neurosurg. (2012) 26:202–7. 10.3109/02688697.2011.626879 22085249

[145] Artru F Chacornac R Deleuze R. Hyperbaric oxygenation for severe head injuries. Eur Neurol. (1976) 14:310–8. 10.1159/000114753 782888

